# Single-Cell Adhesion, Biofilm Formation, and Disinfectant Susceptibility of *Candida albicans* on CAD/CAM-Manufactured Occlusal Splint Resins

**DOI:** 10.3390/antibiotics15100941

**Published:** 2026-09-22

**Authors:** Laura Beatriz Ribeiro Falconi, Larianne de Sousa Moisés, Sabrina Romão Gonçalves Coelho, João Fernando Carrijo Queiroz, Giovanna Gomes Maciel, Lucas Portela Oliveira, Sebastian Aguayo, Ana Carolina Pero

**Affiliations:** 1 Department of Dental Materials and Prosthodontics, School of Dentistry, São Paulo State University (UNESP), Araraquara 14801-385, SP, Brazil; l.falconi@unesp.br (L.B.R.F.); larianne.moises@unesp.br (L.d.S.M.); sabrina.coelho@unesp.br (S.R.G.C.); joao.queiroz@unesp.br (J.F.C.Q.); giovanna.maciel@unesp.br (G.G.M.); 2 Department of Dentistry, Federal University of Juiz de Fora, Governador Valadares Campus (UFJF/GV), Governador Valadares 35020-360, MG, Brazil; lucas.portela@ufjf.br; 3 Institute for Biological and Medical Engineering, Pontificia Universidad Católica de Chile, Santiago 7820436, Chile; sebastian.aguayo@uc.cl; 4 School of Dentistry, Faculty of Medicine, Pontificia Universidad Católica de Chile, Santiago 8331150, Chile

**Keywords:** occlusal splints, CAD/CAM, disinfectant solutions

## Abstract

Occlusal splints used for bruxism prevention can accumulate *Candida albicans* biofilms, highlighting the need to evaluate disinfectant efficacy. Objectives: We aimed to evaluate the nanomechanical properties and nanoadhesion force of *C. albicans* cells and the efficacy of disinfectant solutions on three occlusal splint resins: 3D Bio-Splint (3D-printed), EvoBlock Monocor (milled), and Vipicril (conventional heat-polymerized resin, control). Methods: Circular specimens (10 × 1.2 mm; *n* = 60 per resin for the microbiological assays) were polished, immersed in artificial saliva for 56 h, and subjected to 70 brushing cycles to simulate one week of use. After mature *C. albicans* biofilm formation, specimens were disinfected for 10 min with 0.25% sodium hypochlorite, 2% chlorhexidine, sodium perborate (Corega Tabs), 0.1% PHMB, or PBS (control). CFU/mL counts, XTT assay, and scanning electron microscopy (SEM) analyses were performed. The surface roughness, Young’s modulus, and real-time adhesion of individual *C. albicans* cells onto the resins were measured by employing an atomic force microscopy (AFM) and AFM-based single-cell force spectroscopy (SCFS) with functionalized *C. albicans* probes. Results: The 3D-printed resin was the roughest material. Single-cell adhesion was characterized descriptively: the force and energy distributions obtained on the milled resin were shifted towards lower values in all four cell probes, whereas those obtained on the conventional and 3D-printed resins were similar. For biofilm formation, resin type, disinfection protocol, and their interaction significantly affected outcomes (*p* < 0.001). The printed resin showed the highest microbial load in the control group (63.4 × 10^6^ CFU/mL), whereas the milled resin showed the lowest (3.9 × 10^5^ CFU/mL). Chlorhexidine eliminated CFU counts in all resins, although residual metabolic activity ranged from 10.4% to 40.4%. PHMB on the printed resin showed the greatest discrepancy between residual CFU (4.9% of the control) and cell viability (93.4%), suggesting the persistence of metabolically active cells despite the reduction in cultivable microorganisms. The AFM nanocharacterization showed median Young’s modulus values of 9.92 GPa for the milled resin, 6.44 GPa for the 3D-printed resin, and 4.92 GPa for the conventional resin. Conclusions: The 3D-printed resin showed the highest surface roughness at nanoscale and was more susceptible to microbial colonization than the milled resin and more resistant to the effect of the disinfectants. Chlorhexidine was the most effective disinfectant, since it reduced the CFU to zero and significantly reduced the metabolic activity of *C. albicans* for all the occlusal splint resins.

## 1. Introduction

Occlusal stabilization splints are widely and commonly used in dentistry, mainly for the management of sleep bruxism and the treatment of temporomandibular disorders (TMD), presenting satisfactory results as a means of protecting the teeth and other structures of the stomatognathic system [1,2,3,4,5].

With the advent of the digital workflow and Computer-Aided Design/Computer-Aided Manufacturing (CAD/CAM) systems in dentistry, the conventional method for manufacturing these splints has been replaced by additive manufacturing (3D printing) or subtractive manufacturing (milling) [6]. These methods ensure greater speed, simplicity, and high precision, in addition to enabling the reproduction of multiple devices with complex geometries, thereby providing advantages for both dentists and patients [7,8].

Occlusal stabilization splints used for the management of bruxism are worn, on average, for eight hours per day during sleep, representing a favorable substrate for bacterial adhesion and subsequent biofilm formation by microorganisms present in the oral cavity. Continuous biofilm formation leads to the development of mature dental plaque, which becomes difficult to remove without mechanical intervention. Thick biofilm accumulation on the surface of oral devices may cause gingival irritation associated with increased biofilm formation and may also increase the risk of oral diseases, such as dental caries and oral candidiasis [9,10,11,12].

The human oral cavity harbors a complex microbiome composed of bacteria, fungi, viruses, and protozoa, in which the most prevalent microorganisms include genera such as *Streptococcus*, *Haemophilus*, *Neisseria*, *Porphyromonas*, *Prevotella*, and *Veillonella*, while *Candida albicans* is the most prevalent fungal species [13]. *C. albicans* exhibits a remarkable ability to interact with other oral microorganisms, which largely determines its virulence and its capacity to develop resistance to antimicrobial therapies, thereby increasing disease severity and the risk of developing systemic infections that are difficult to treat [14,15,16,17,18]. Several studies have demonstrated the interaction between *Candida albicans* and *Streptococcus mutans*, associating this fungus with the etiology and increased virulence of dental caries [13,19,20,21].

The adhesion of microorganisms on resin surfaces is influenced by many factors, such as surface roughness, surface charge, surface free energy (SFE), hydrophobicity/hydrophilicity, composition of the biomaterial, and interactions among microorganisms [22,23,24]. Wuersching et al. [12] demonstrated a direct correlation between surface roughness and the number of viable bacteria present in mixed-species biofilms formed on materials used for occlusal splints and orthodontic aligners. Therefore, the implementation of an effective hygiene protocol for biofilm removal from occlusal splints is essential. Hygiene methods include mechanical approaches, such as manual brushing and ultrasonic cleaning; chemical approaches, including immersion in cleansing agents, disinfectants, and mouthwashes; and physical approaches, such as microwave irradiation, low-level laser therapy, and photodynamic therapy [25,26]. To optimize hygiene efficacy, the combination of mechanical methods with chemical agents is recommended for biofilm removal from prosthetic devices and occlusal splints [25].

Immersion in aqueous solutions, such as sodium hypochlorite, chlorhexidine digluconate, and sodium perborate-based effervescent tablets, has been widely used as a chemical method for disinfecting these devices by promoting the removal of microorganisms [7,27,28,29,30,31,32]. Mayta et al. [33] showed that chlorhexidine 0.12%, Corega Tabs, saliva, and distilled water influenced the surface roughness, hardness, flexural strength, and flexural modulus of 3D-printed occlusal splint resins.

A promising and innovative alternative for biofilm removal in dentistry is the use of polyhexamethylene biguanide (PHMB), an amphipathic cationic polymer with broad-spectrum antimicrobial properties and well-established biological safety [34]. PHMB has been widely used as a disinfectant in the medical field and shows considerable potential for cleaning intraoral prosthetic devices because of its low tissue toxicity and effectiveness against mixed and complex biofilms [35], in addition to its low cost and widespread availability. A systematic review that evaluated the effects of different antimicrobial substances in the oral environment demonstrated that mouthwashes containing PHMB preserve the stability of the oral microbiota, maintaining efficacy in the control of dental plaque and inflammation [36]. PHMB has also shown positive results in other clinical applications, such as an intracanal irrigant, promoting a significant reduction in microbial load, including against *Candida albicans* [37,38].

To prevent the development of oral pathologies, a highly relevant aspect is understanding how microorganisms interact and attach to biomaterials, as this is the initial, crucial step for biofilm formation [39,40,41,42]. In this regard, atomic force microscopy (AFM) based approaches—such as single-cell force spectroscopy (SCFS)—have emerged as highly versatile techniques to explore real-time cellular adhesion of microbes to biomaterials under physiological conditions with sub-cellular sensitivity [43,44]. Recent investigations have utilized AFM and SCFS to explore the biology of *C. albicans* [45], and in this context, it becomes reasonable to investigate if *C. albicans* adhesion at the single-cell level is determined by the surface properties of occlusal splint materials obtained by CAD-CAM.

Considering that the use of occlusal splints is quite common in the treatment of sleep bruxism and in the prevention of TMDs, and that the properties of the materials used in the manufacture of occlusal splints obtained by digital workflow are still little explored in the literature, this study has the potential to identify future strategies in the prevention of oral pathologies in users of occlusal splints. The aim of this study was twofold: first, to characterize descriptively, and at the level of the individual cell, the nanomechanical properties of three occlusal splint resins and the distributions of the adhesion forces developed by *C. albicans* on each of them; second, to compare the effectiveness of different disinfectant solutions in reducing *C. albicans* biofilm, while also evaluating the surface topography of these materials after being exposed to brushing and the disinfectant protocols for the simulated 1-week period. The null hypothesis tested was that neither the type of resin nor the disinfectant solution would affect the microbial load or the metabolic activity of *C. albicans* biofilms formed on occlusal splint materials. The nanomechanical characterization is presented descriptively, and no hypothesis is tested on those outcomes.

The findings demonstrated that the milled resin was the least susceptible and the 3D-printed resin the most susceptible to microbial colonization. The 3D-printed resin, the roughest material, exhibited the highest microbial adhesion capacity, characterized by greater adhesion force and energy, more rupture events per curve, and longer rupture lengths. This result suggests an association between higher nanoscale surface roughness and stronger, more persistent adhesion interactions, which have direct implications for biofilm formation on occlusal splints.

Chlorhexidine was the most effective disinfectant in reducing colony-forming units (CFUs), whereas all disinfectant solutions significantly reduced metabolic activity, except PHMB for the 3D-printed resin. The discrepancy between CFU counts and XTT assay results, particularly for the PHMB-treated 3D-printed resin, may have several possible explanations, including the presence of viable but non-culturable cells. However, this possibility cannot be directly confirmed by the CFU and XTT assays performed in this study. These findings indicate that the antimicrobial response may be influenced by the substrate and the experimental conditions.

## 2. Results

### 2.1. Surface Topography and Roughness

Roughness distinguished the three materials descriptively (Figure 1). Values are reported as the median and range of the four quantitative-imaging maps acquired per material. The 3D-printed resin was the roughest (median Ra = 814 nm, range 559–1119 nm; median RMS = 1006 nm, range 744–1313 nm), the milled material was the smoothest (median Ra = 236 nm, range 173–267 nm; median RMS = 318 nm, range 226–330 nm), and the conventional material was intermediate (median Ra = 350 nm, range 321–428 nm; median RMS = 479 nm, range 392–550 nm).

### 2.2. Young’s Modulus

Young’s modulus was likewise summarized descriptively across the four quantitative-imaging maps acquired per material. The milled resin was the stiffest (median = 9.92 GPa, range 8.60–11.35 GPa), followed by the 3D-printed resin (median = 6.44 GPa, range 6.01–7.27 GPa) and the conventional resin (median = 4.92 GPa, range 4.02–5.44 GPa).

The complete distribution of all points from the surface indentation map (58,442 points per milled resin, 46,341 points per 3D-printed and 44,440 points per conventional surface) is presented as a histogram (Figure 2). The histogram reveals the mechanical heterogeneity within each resin—a feature not captured by the median summary.

The per-probe distributions of maximum adhesion force are shown in Figure 3. The median values were 0.29 nN for the milled resin, 0.86 nN for the conventional resin and 1.22 nN for the 3D-printed resin. The histogram shows that, for the milled resin, almost all interactions are concentrated at low force levels, whereas the distributions for the conventional and 3D-printed resins are broader, featuring a tail of strong adhesion events.

Adhesion energy, representing the total interaction energy measured between the *C. albicans* cell and substrate during retraction (area under the force curve), followed the same pattern, as shown in Figure 4. The median values were 16.2 aJ for the milled group, 54.0 aJ for the conventional group and 76.9 aJ for the 3D-printed group.

Besides the whole-cell adhesion parameters of maximum adhesion force and energy, the force required to break each individual receptor-surface bond was also examined. The median values were 372 pN (milled), 656 pN (conventional), and 725 pN (3D-printed). Figure 5 shows that the peak position of the per-probe distributions is similar across the materials. What differs most is the extent of the tail of strong bonds, which is more pronounced for the 3D-printed and conventional resins.

On the other hand, the rupture length of these per-probe interactions, shown in Figure 6, represents the stretching distance of the molecules until rupture, and was greater for the 3D-printed material (median = 127 nm) than for the conventional (88 nm) and milled (82 nm) materials, whose distributions were similar to each other.

Finally, the number of rupture events per curve reflects how many molecular anchor points were formed during each interaction are illustrated in Figure 7. The milled resin shows approximately 70% of curves with zero events indicating weak, sparse adhesion, whereas the conventional and 3D-printed resins exhibit a higher proportion of curves with multiple anchor points.

### 2.3. Biofilm Formation

For both assays (CFU/mL and XTT), the same significant effect was observed for resin type (CFU: *F*(2, 75) = 289.23, *p* < 0.001; XTT: *F*(2, 75) = 73.09, *p* < 0.001), showing that microbial load and metabolic activity were higher for the 3D-printed resin, intermediate for the conventional resin, and lower for the milled resin. The three resins differed from one another in both assays: for the 3D-printed resin versus the conventional resin, the 3D-printed resin versus the milled resin and the conventional resin versus the milled resin, *p* < 0.001 in the CFU counts and *p* < 0.001 in the XTT readings.

The statistical analysis of the microbial load (CFU/mL) showed a good fit to the data, explaining 97.7% of the observed variability (*R*^2^ = 0.977). The effect of the disinfectant solution was significant (*F*(4, 75) = 554.84, *p* < 0.001). Chlorhexidine was the most effective, it reduced the count to zero for all resins and differed from all the others (versus the control, hypochlorite, PHMB and Corega Tabs (perborate), *p* < 0.001 in every case), followed by PHMB, which was superior to hypochlorite and Corega Tabs (perborate) (*p* < 0.001 in both comparisons). Hypochlorite and Corega Tabs (perborate) also reduced the count relative to the untreated control (*p* < 0.001 in both comparisons). The only pair that did not differ significantly was hypochlorite vs. Corega Tabs (*p* = 0.255).

The significant interaction between disinfectant solution and resin type (*F*(8, 75) = 42.33, *p* < 0.001) indicated that the effect of the disinfectant solutions on the evaluated response varied according to resin type, as shown in Figure 8. PHMB reduced the count to zero (similar to chlorhexidine, *p* = 1.000) for the milled resin, something that did not occur with the conventional and printed resins (PHMB on the milled resin versus PHMB on the conventional resin and versus PHMB on the 3D-printed resin, *p* < 0.001 in both comparisons).

The biofilm metabolic activity analysis (XTT) explained 75.8% of the observed variability (*R*^2^ = 0.758). The effect of the disinfectant solution was significant (*F*(4, 75) = 13.20, *p* < 0.001). Chlorhexidine reduced viability compared to the control (*p* < 0.001) but did not differ from Corega Tabs (perborate) (*p* = 1.000), hypochlorite (*p* = 0.833), or PHMB (*p* = 0.511). Hypochlorite, PHMB and Corega Tabs (perborate) also reduced viability relative to the control (*p* < 0.001 in every case) and did not differ from one another (hypochlorite versus PHMB, *p* = 0.983; hypochlorite versus Corega Tabs, *p* = 0.885; PHMB versus Corega Tabs, *p* = 0.587). The significant interaction between disinfectant solution and resin type (*F*(8, 75) = 4.48, *p* < 0.001) indicated that the effect of the disinfectant solutions on the evaluated response varied according to resin type, as shown in Figure 9. For the printed resin in PHMB, metabolic viability remained at ~93%, practically the same as the control (*p* = 1.000) and well above the results observed with PHMB in the other resins (versus PHMB on the conventional resin and versus PHMB on the milled resin, *p* < 0.001 in both comparisons).

The comparative analysis between the CFU and XTT assays (Figure 10), normalized to their respective controls, showed that biofilm metabolic activity was generally higher than the cultivable microbial load under all or most of the experimental conditions.

### 2.4. Surface Topography

Scanning electron microscopy (SEM) images of the Candida albicans biofilm formed on conventional, 3D-printed, and milled resins, obtained at 1000× magnification (one micrograph at 1200×, as indicated in the figure) after treatment with different disinfectant solutions, are shown in Figure 11.

## 3. Discussion

The null hypothesis was rejected, as both resin type and disinfectant solution significantly influenced the microbial load and the metabolic activity of the *Candida albicans* biofilm formed on the occlusal splint materials. The nanomechanical characterization, which is presented descriptively, showed that the three materials differed in surface roughness and stiffness, and that the distributions of single-cell adhesion forces differed between them in position and spread.

Surface roughness and Young’s modulus results showed that the 3D-printed resin exhibited the roughest surface and stiffness comparable to the other resins. The systematic review and meta-analysis published by Valenti et al. [46] concluded that the mechanical performance of 3D-printed materials for oral appliances was similar to that of conventional resins, while milled splint materials did not perform better than conventional heat-cured acrylic resin.

The microbiological assays showed consistent behavior, demonstrating that resin type influenced both the cultivable microbial load and the biofilm metabolic activity. In both outcomes, the 3D-printed resin exhibited the highest CFU and metabolic activity values, followed by the conventional and the milled resins. The findings of the present study corroborate the results of Freitas et al. [47], who observed that 3D-printed specimens exhibited greater surface roughness, which favored microbial adhesion, and lower flexural strength than the milled resin. Similarly, Alfouzan et al. [48] found that 3D-printed denture base resin showed greater biofilm formation, followed by the conventional thermopolymerized resin, whereas the milled discs showed the lowest CFU/mL count among all the materials evaluated. In contrast, Schubert et al. [49] found that the adhesion of *C. albicans* was greater on the occlusal splints fabricated by milling and 3D printing compared with those produced by the conventional thermoforming and pressing methods.

The influence of resin types on the microbial load and the biofilm metabolic activity suggests that the surface characteristics of the materials played a decisive role in biofilm adhesion and development. Among many factors, including the substrate properties, such as chemical composition, surface free energy, surface charge, hydrophobicity/hydrophilicity, and interactions among microorganisms, it is well stablished that surface roughness influences the initial adherence of microorganisms to the substrate and consequent biofilm formation [50,51,52]. However, in this study, the surface roughness was standardized to Ra ≤ 0.2 µm, to simulate the polished surface of the occlusal splint, according to the threshold for microbial adhesion [53]. Thus, the influence of surface roughness on adhesion of *C. albicans* could be demonstrated by the SCFS analyses, even though the microscale roughness was standardized. AFM results regarding roughness showed marked differences between the resins at the nanoscale (Figure 1). The 3D-printed resin exhibited the highest surface roughness in comparison to the milled material, and the conventional resin showed intermediate Ra and RMS values.

Additionally, the arrangement of layers and the gaps between them on the surfaces produced by 3D printing make these surfaces more prone to microbial accumulation [32,40,54,55]. Nevertheless, it remains interesting that at the individual *C. albicans* cell level, there is still cell-to-cell variability in the behavior of each AFM microbial probe across the three resins for adhesion force (Figure S1), adhesion energy (Figure S2), rupture length (Figure S3), and breaking forces (Figure S4). However, this behavior is to be expected, as previous research has shown that at the individual single-cell level there are population-based differences in adhesion capabilities, probably due to variations in available microbial adhesins, growth states, or other biological factors [45,56,57].

Despite the different behavior of microbial adhesion across the different occlusal splint resins, all disinfection protocols promoted biofilm reduction. Chlorhexidine showed the greatest reduction in cultivable microbial load, achieving complete elimination of CFU for all resins. Chlorhexidine is considered an effective chemical disinfectant for denture hygiene, widely used for biofilm control and the prevention of denture stomatitis. Its ability to adsorb onto oral surfaces and to be gradually released allows the effective reduction in the initial adhesion of fungi and other microorganisms, consequently minimizing biofilm formation [58].

It was also observed that, on the milled resin, immersion in PHMB completely eliminated the CFU of *C. albicans*. This performance was equivalent to that of chlorhexidine. In contrast, this effect was not observed on the conventional and 3D-printed resins. This finding shows that the efficacy of the same disinfectant may vary according to the characteristics of the material on which it acts. A reasonable explanation for these findings is based on the AFM results. The distributions of adhesion forces obtained on the milled resin were displaced towards lower values than those obtained on the conventional and 3D-printed resins, in every cell probe examined. The same was observed for the adhesion energy, which represents the total energy of the interaction between the *C. albicans* cells and the surfaces, even though the central distribution was similar across the materials. Similar results were found for single unbinding rupture forces and distances. The results of surface roughness and SEM images also corroborate these findings, since the milled resins showed smoother surfaces and lower occurrence of filamentous structures of *C. albicans*, known to be more virulent forms with higher expression of adhesion factors [59]. The polymorphism of *Candida* spp., which includes yeast, pseudohyphal, and hyphal forms, constitutes an important virulence factor. Among these forms, the hyphal form is considered the most pathogenic because of its ability to exert mechanical force, penetrate the host epithelial tissues, cause endothelial damage, and facilitate the dissemination of the infection through the bloodstream [60]. However, the SEM images were strictly for descriptive purposes, since only two specimens were analyzed per experimental condition. In addition, virulence and hyphal formation were not assessed in this study.

Thus, the results describe an association between surface roughness and microbial adhesion, in which the rougher acrylic surfaces were accompanied by adhesion force distributions displaced towards higher values, which may have direct implications for biofilm formation on occlusal splints.

In contrast, in this study, the XTT assay indicated that all disinfectants promoted similar reductions in metabolic activity, except PHMB on the 3D-printed resin. For this material, PHMB reduced the CFU counts, while relatively high metabolic activity of *C. albicans* was observed. The dissociation between the CFU and XTT results may reflect the persistence of metabolically active cells that were not recovered under the culture conditions used. However, the mechanisms underlying this discrepancy cannot be determined from the CFU and XTT assays. Previous studies have suggested that PHMB may affect cell membrane integrity and biofilm structure [59,60,61], which could potentially contribute to the observed response. According to Carvalho et al. [62], the antimicrobial efficacy of PHMB may vary according to the microbial strain, which may help explain the maintenance of metabolic activity observed in the present study and the differences between CFU and XTT measurements.

The low effectiveness of PHMB on the metabolic activity of *C. albicans*, which was significatively observed for the 3D-printed resin, may also be associated with the SEM images, that revealed a greater predominance of filamentous structures of *C. albicans* on the 3D-printed resins. These findings corroborate the AFM results of surface roughness, suggesting that the surface characteristics of the material may influence the morphological transition of *C. albicans* and, consequently, its virulence potential [60]. Thus, the results suggest that the antimicrobial potential of PHMB depends not only on the target microorganism but also on the environment and the surface to which it is applied.

In this study, Corega Tabs showed a similar effect to that of sodium hypochlorite on the microbial load (CFU), irrespective of the occlusal splint resin. Bento et al. [63], also demonstrated that Corega Tabs significantly reduced the microbial colonization of *C. albicans* on 3D-printed and milled denture base resins. The good performance of Corega Tabs in reducing the microbial load of *C. albicans* from the 3D-printed resin, despite the stronger adhesion parameters obtained from the AFM results for this material, may be attributed to the effervescent reaction of the tablet when dissolved in water, releasing oxygen and promoting mechanical removal from the surface [64,65].

The main limitations of this in vitro study were the inability to simulate the full complexity of the oral cavity environment, where factors such as temperature and the salivary pellicle could influence these results, as well as the use of a single-species biofilm model and a relatively short experimental period. In addition, only one commercial brand of each type of material was tested. Furthermore, two different strains were used for the single-cell adhesion assays and the biofilm assays, which limits a straight correlation from these results. SEM analysis provided primarily qualitative morphological observations, with limited quantitative assessment.

Another experimental limitation concerns the SCFS setup used to study single-cell-level adhesion of *C. albicans* onto splint materials. Firstly, these experiments yield large numbers of cell–substrate interactions across regions of interest; therefore, variations in surface heterogeneity can lead to variability in the resulting adhesion data, particularly on more irregular topographies such as acrylics. Secondly, cells within the same population can still display important differences in their adhesive behavior across biological replicates, and thus it remains challenging to determine significant differences across all the studied conditions. These outcomes are therefore reported descriptively throughout, and no inferential conclusion is drawn from them. Nevertheless, future studies are necessary to elucidate the influence of different occlusal splint resins on single-cell microbial adhesion and disinfectant efficacy using multispecies and longer-term biofilm models, multiple materials within each manufacturing category, the use of controlled roughness gradients, larger sample sizes, and more comprehensive quantitative analyses, as well as to confirm these results in both in situ and clinical studies.

Overall, the main finding of this study was that the nanomechanical characterization of the material surface yielded results that were extremely relevant to the interpretation of the results, showing that the highest surface roughness at the nanoscale and the increased early attachment was associated with the *C. albicans* nanoadhesion on 3D-printed resins. All disinfectant solutions promoted a reduction in biofilm metabolic activity, except PHMB, particularly on 3D-printed resins, which proved highly resistant to microbial reduction. Since this is an in vitro and short-term study, the disinfectant effectiveness of chlorhexidine in reducing CFU counts should be interpreted under the experimental conditions tested.

## 4. Materials and Methods

### 4.1. Experimental Groups

Disc-shaped specimens (10 mm in diameter and 1.2 mm in thickness) [66] were fabricated from PriZma 3D Bio Splint resin (Makertech Labs, São Paulo, SP, Brazil), EvoBlock Monocor milling blocks (Evoden Produtos Odontológicos, Pirassununga, SP, Brazil), and Vipicril heat-polymerized acrylic resin (Dental Partner, São Paulo, SP, Brazil). On each of three independent occasions, 20 specimens were prepared per material and randomly allocated into five experimental groups, with four specimens per group, according to the disinfectant solution used: 0.25% sodium hypochlorite, 2% chlorhexidine, Corega Tabs, 0.1% polyhexamethylene biguanide (PHMB), and phosphate-buffered saline (PBS) as the negative control.

Within each disinfectant group, two specimens were assigned to colony-forming unit counting and two to the XTT assay on each occasion, so that over the three occasions, six specimens per material and disinfectant combination were evaluated in each assay, totalling 60 specimens per material for the microbiological experiment. In the XTT assay, each of these six specimens was read in duplicate aliquots.

For surface topography analysis by scanning electron microscopy (SEM), one additional independent experiment was performed in duplicate, providing two specimens per material and disinfectant combination that were used exclusively for this purpose, as SEM is a destructive technique requiring carbon coating of the specimens prior to analysis.

The atomic force microscopy measurements were performed on separate specimens and were not allocated to the disinfectant groups: four quantitative-imaging maps were acquired per material for topography, roughness and Young’s modulus (Section 4.3), and four *C. albicans* cell probes were used for the single-cell force spectroscopy, with each probe being contacted with all three materials (Section 4.4).

### 4.2. Specimen Fabrication

#### 4.2.1. Fabrication of 3D-Printed Resin Specimens

Specimens were fabricated using a Flashforge Foto 6.0 printer (Zhejiang Flashforge 3D Technology Co., Jinhua, Zhejiang, China). The virtual design of the specimens was created using Adobe Meshmixer v. 3.5 (Autodesk Inc., San Rafael, CA, USA) and subsequently exported as an STL file. The file was then imported into the proprietary software (FlashDLPrint v. 3.28.0), where the printing orientation was set at 90° relative to the build platform.

Following model preparation and slicing, the 3D Bio Splint resin was homogenized and poured into the printer resin tank. Specimen polymerization was initiated by ultraviolet light (LED panel, λ = 405 nm). Printing was performed according to the manufacturer’s recommendations (e.g., layer exposure time, interval, and energy settings), using a layer thickness of 50 µm. After printing, the specimens were washed in 99% isopropyl alcohol for 5 min to remove residual surface monomer [56] and subsequently post-cured using 405 nm ultraviolet light in a cyclOne post-processing unit (3D Washing and Post-Curing Station, dOne 3D, Ribeirão Preto, SP, Brazil) for 10 min, according to the resin manufacturer’s recommendations (5 min on each side) [67]. Prior to testing, the specimens were ultrasonically cleaned for 1 min in distilled water containing 1% detergent, dried with absorbent paper, and stored in distilled water at 37 °C for 50 ± 2 h [68].

#### 4.2.2. Fabrication of Milled Resin Specimens

Specimens were fabricated from pre-polymerized PMMA EvoBlock Monocor resin blocks (Evoden Produtos Odontológicos, Pirassununga, SP, Brazil) using a Coritec 150i PRO CAD/CAM milling machine (imes-icore GmbH, Eiterfeld, Germany). The blocks were milled to obtain circular specimens measuring 10 mm in diameter and 1.2 mm in thickness. After milling, the specimens were finished to remove any excess material and then stored in distilled water at 37 °C for 50 ± 2 h prior to testing [68].

#### 4.2.3. Fabrication of Vipicril Resin Specimens

Vipicril heat-polymerized acrylic resin specimens were fabricated using a stainless-steel mold containing ten circular cavities measuring 10 mm in diameter and 1.2 mm in thickness. The mold was invested in a metal flask using Type III dental stone over a glass slab. After the stone had set, a second frosted glass slab was positioned over the mold/stone assembly, and the counterflask was filled with an additional portion of Type III dental stone, completing the investing procedure.

After flask separation, the mold surfaces were isolated with Cel-Lac separating medium, and the Vipicril acrylic resin was packed into the mold. The resin was mixed at a powder-to-liquid ratio of 3:1 (*v*/*v*), according to the manufacturer’s instructions. The polymer powder was weighed using a precision electronic balance, while the monomer liquid was measured with a pipette.

Compression molding was carried out in two stages using a hydraulic press. Initial pressing was performed under a load of 0.5 ton-force. The flask was then opened, and excess resin was removed using a Le Cron instrument. Final pressing was subsequently performed under sequential loads of 0.5, 0.75, and 1.25 ton-force.

The flask remained under pressure for 30 min to allow adequate resin flow and proper diffusion of the monomer into the polymer matrix [69]. After this period, the flasks were secured with flask clamps and polymerized using an automatic water-bath polymerization unit (SL-150/30; Solab Equipamentos para Laboratórios, Piracicaba, SP, Brazil) following the manufacturer’s recommended curing cycle of 1.5 h at 73 °C followed by 30 min at 100 °C. After polymerization, the flasks were allowed to cool at room temperature overnight before deflasking. The specimens were then finished to remove excess resin. One surface of each specimen, corresponding to the polished surface of an occlusal splint, was polished using silicon carbide abrasive papers (220-, 400-, 600-, and 1200-grit) for 30 s each to standardize the surface roughness to Ra ≤ 0.2 µm [51]. After polishing, the mean surface roughness (Ra, µm) was measured with a portable digital profilometer (SJ-400, Mitutoyo Corporation, Kawasaki, Kanagawa, Japan; accuracy: 0.01 µm), over a 2.4 mm tracing length at a stylus speed of 0.5 mm/s using a 5 µm stylus tip radius. All specimens were stored in distilled water at 37 °C for 50 ± 2 h prior to testing [68].

### 4.3. Surface Roughness and Young’s Modulus

Topographic and surface roughness analysis of the acrylic substrates was carried out with the use of a JPK Nanowizard V (Bruker Nano GmbH, Berlin, Germany) AFM mounted on a Zeiss Axio Observer Microscope (Carl Zeiss Microscopy GmbH, Jena, Germany). Briefly, 50 × 50 µm scans with 256 × 256 pixels were obtained with advanced Quantitative Imaging (Advanced QI) mode across three distinct regions of each acrylic surface, yielding four quantitative-imaging maps per material, by employing SCOUT 350 RAu probes (NuNano Ltd., Bristol, UK; nominal spring constant = 42 N/m), with the analysis done in the medium air. From the resulting scans, height, stiffness, and 3D reconstruction images were obtained for the different substrates in the JPKSPM Data Processing software v.8.1.89 (JPK Instruments, Berlin, Germany), and Young’s modulus was calculated by fitting the Sneddon model to each individual force curve from the QI force maps. The Ra and RMS roughness parameters were also calculated from the scans using the same software across the same four maps per material. These four maps constitute the replicates of the roughness and Young modulus outcomes, which are reported descriptively (Section 4.8).

### 4.4. Fabrication of C. albicans Cell Probes for Single-Cell Force Spectroscopy (SCFS) with Atomic Force Microscopy (AFM)

For all SCFS experiments, a JPK Nanowizard V (Bruker Nano GmbH, Berlin, Germany) AFM mounted on a Zeiss Axio Observer Microscope (Carl Zeiss Microscopy GmbH, Jena, Germany) was employed. Tipless silicon nitride cantilevers (QUEST R 200 TL, NuNano Ltd., Bristol, UK; nominal spring constant = 0.2 N/m) were individually calibrated for sensitivity and spring constant and coated with 0.1% poly-L-lysine (PLL) immediately before experiments, using a previously published approach [69]. Briefly, 20 µL of the PLL solution was carefully deposited directly onto the cantilever, incubated for 5 min, and the excess solution was gently removed using a micropipette tip. The cantilevers were subsequently allowed to air-dry, dried under a gentle N2 stream, and immediately used for *C. albicans* immobilization.

A laboratory strain of *Candida albicans* (ATCC 90028) was reactivated in Tryptic Soy Broth (TSB) and incubated at 37 °C for 48 h under aerobic conditions. The ATCC 90028 strain was used because it is the type typically used in AFM studies and grows predominantly in the yeast form, making it feasible to functionalize the probe with a single cell. Following incubation, a single colony of cells were suspended in 1 mL of sterile phosphate-buffered saline (PBS) and manually homogenized. A 50 µL droplet of the diluted suspension was then transferred onto a clean glass slide and examined using the optical microscope (20× objective) to identify isolated fungal cells. The PLL-coated cantilever was then carefully approached to an isolated *C. albicans* cell under optical guidance and placed in contact with the cell at a low setpoint (<0.5 nN) for 1 min, allowing stable attachment. Immobilization of the *C. albicans* cell was confirmed optically upon cantilever retraction.

Subsequently, the functionalized AFM probes were transferred atop the acrylic resin specimens (immobilized to a glass slide with double-sided tape), being careful to ensure constant hydration throughout the process. Adhesion measurements were performed in PBS buffer in force spectroscopy mode, employing four independent *C. albicans* cell probes (*n* = 4), each of which was contacted sequentially with all three acrylic surfaces so that every cell served as its own control across materials, using a setpoint force of 1 nN, a Z-length of 1.0 µm, a Z-speed of 2.0 µm/s, and a contact time of 1 s. For each cell probe and each acrylic surface, force–distance curves were acquired within 10 × 10 µm areas across distinct regions of the specimen. After quality filtering, between 1922 and 2748 force curves remained per parameter, distributed across the four cell probes and the three surfaces. The individual cell probe was defined as the experimental unit of the single-cell force spectroscopy experiments.

The resulting force–distance curves were processed using the JPKSPM Data Processing software v.8.1.89 (JPK Instruments, Berlin, Germany). Force curves with no surface contact or where the baseline was not reached after full retraction were excluded from analysis. From the resulting curves, the maximum adhesion force (nN), adhesion energy (aJ), and the number of individual unbinding events and their rupture length (nm) and breaking force (nN) were extracted and tabulated. Data are shown both aggregated and individualized for each biological replicate (cell probe) across all variables.

### 4.5. Simulated Nocturnal Use and Toothbrushing

In this study, nocturnal use of the occlusal splint was simulated over a period of 1 week (8 h per night; 56 h over 7 days). The occlusal splints were also subject to 70 brushing cycles to simulate the same one-week period. Previous studies [66,67] reported that one year of brushing corresponds to 3650 cycles and assumed that a dental surface is brushed 20×/day or 10 cycles/day. Thus, 70 brushing cycles would simulate one week of brushing. These simulations were performed prior to microbiological evaluations.

For simulation of nocturnal use, each specimen was individually immersed in 5 mL of artificial saliva in a 24-well plate and incubated at 37 °C for a total of 56 h (equivalent to seven nights of 8 h use). Artificial saliva was prepared by a compounding pharmacy and contained KCl (0.4 g/L), NaCl (0.4 g/L), CaCl_2_·2H_2_O (0.906 g/L), NaH_2_PO_4_·2H_2_O (0.690 g/L), Na_2_S·9H_2_O (0.005 g/L), and urea (1 g/L), adjusted to pH 6.5. The artificial saliva was renewed every 8 h, totaling seven solution changes.

Toothbrushing simulation was performed using a six-station brushing machine. The apparatus consisted of a central motor connected to two primary metallic arms, each driving three secondary arms. Soft-bristle toothbrushes (Sorriso Soft, Colgate-Palmolive Ind. e Com. Ltd.a., São Paulo, SP, Brazil) were attached to the secondary arms. The removable specimen holders were filled with a slurry containing distilled water and dentifrice (Colgate, Colgate-Palmolive, São Bernardo do Campo, SP, Brazil) at a 1:1 weight ratio in sufficient volume (200 g) to completely immerse the specimens. The holders contained individual slots to securely position the specimens during brushing. Each specimen underwent a total of 70 brushing cycles, corresponding to twice-daily brushing for one week.

Following brushing, the specimens were ultrasonically cleaned for 1 min in distilled water containing 1% detergent, dried with absorbent paper, and disinfected by ultraviolet light exposure for 20 min on each surface before the biological assays [32].

### 4.6. Microbiological Evaluation

#### 4.6.1. Microorganism and Culture Conditions

A reference strain SC5314 of *Candida albicans* (ATCC MYA-2876) was thawed and reactivated on Sabouraud Dextrose Agar (SDA) at 37 °C for 48 h. Following reactivation, a pre-inoculum was prepared by transferring five isolated colonies into Tryptone Yeast Extract (TYE) broth supplemented with 1% glucose and incubating overnight (16–18 h) at 37 °C.

The fungal suspension was subsequently diluted 1:10 in fresh TYE broth supplemented with 1% glucose and incubated again at 37 °C until the optical density at 540 nm (OD_540_) reached the mid-exponential growth phase, according to the previously established growth curve. At this stage, the suspension contained approximately 6.5 × 10^6^ CFU/mL.

#### 4.6.2. Mature Biofilm Formation

The resin specimens were placed individually into 24-well plates. Each well received 450 µL of the fungal suspension adjusted to 1 × 10^7^ CFU/mL and 450 µL of TYE broth supplemented with 1% glucose, resulting in a final volume of 900 µL. The plates were incubated on an orbital shaker at 75 rpm and 37 °C for 90 min to allow fungal adhesion.

Following the adhesion phase, the specimens were removed, gently rinsed once with phosphate-buffered saline (PBS) to remove non-adherent cells, and transferred to new 24-well plates containing 900 µL of fresh TYE broth supplemented with 1% sucrose. The specimens were then incubated on an orbital shaker (75 rpm, 37 °C) for biofilm development. After 24 h, the culture medium was replaced with fresh medium to promote biofilm maturation. After a total incubation period of 48 h, the mature biofilms were gently washed once with PBS to remove non-adherent cells.

The experiment was performed on three independent occasions, with two specimens per experimental condition assigned to colony-forming unit (CFU/mL) counting and two to the XTT metabolic activity assay on each occasion, and on one additional independent occasion in duplicate for scanning electron microscopy (*n* = 2).

After biofilm formation, specimens from each resin material were randomly assigned (Microsoft Excel; Microsoft Corp., Redmond, WA, USA) to the different disinfectant treatment groups according to the immersion protocols.

#### 4.6.3. Immersion of Specimens in Disinfectant Solutions

Following an additional rinse with phosphate-buffered saline (PBS) to remove non-adherent cells, the specimens were individually transferred to 24-well plates and maintained at room temperature. Each well received 900 µL of the corresponding disinfectant solution. Regardless of the disinfectant used, immersion was performed for 10 min, simulating one weekly disinfection cycle. This short-term protocol investigated the effectiveness of each disinfectant used once [70].

The sodium perborate solution (Corega Tabs; Stafford Miller Ind., Rio de Janeiro, RJ, Brazil) was prepared according to the manufacturer’s instructions by dissolving one tablet in 200 mL of water heated to 40 °C in a covered glass container [32]. The solution temperature was monitored using a calibrated thermometer.

During the immersion period, the 24-well plates containing the specimens and disinfectant solutions were maintained inside a laminar flow cabinet.

#### 4.6.4. Colony-Forming Unit (CFU/mL)

Following the disinfection protocols, the specimens were transferred to new 24-well plates containing 900 µL of PBS. The residual mature biofilm adhered to the specimen surface was mechanically detached by scraping with a sterile pipette tip for 1 min. Subsequently, 100 µL of the resulting biofilm suspension was transferred to a microcentrifuge tube containing 900 µL of PBS to initiate tenfold serial dilutions.

Aliquots from the serial dilutions were plated onto Sabouraud Dextrose Agar (SDA) supplemented with chloramphenicol and incubated at 37 °C for 48 h. The number of colony-forming units per milliliter (CFU/mL) was calculated using the following formula: CFU/mL = (number of colonies × 10^n^)/q; here, n corresponds to the absolute value of the dilution (1, 2, 3 or 4), and q represents the volume (0.025 mL) pipetted for each dilution during plate seeding. CFU/mL values were expressed in scientific notation, and the arithmetic mean of the duplicates was calculated. The resulting data were transformed using the formula log(CFU + 1)/mL.

#### 4.6.5. Cell Metabolic Activity (XTT Assay)

Biofilm metabolic activity was evaluated using the XTT reduction assay. The XTT solution [2,3-bis(2-methoxy-4-nitro-5-sulfophenyl)-5-[(phenylamino)carbonyl]-2H-tetrazolium hydroxide] was prepared at a concentration of 2.5 mg/mL in glucose-supplemented PBS and stored at −80 °C until use. Menadione (0.4 mM in acetone) was freshly prepared immediately before each experiment.

After rinsing the specimens with PBS, they were transferred to new 24-well plates. Subsequently, 500 µL of the XTT working solution, consisting of 5 mL of XTT solution supplemented with 1.5 µL of menadione, was added to each well. The plates were incubated in the dark at 37 °C for 3 h.

Following incubation, duplicate aliquots of 200 µL from each well were transferred to a 96-well microplate. The two aliquots are technical replicates of the same specimen; their average absorbance represented the metabolic activity of that specimen and was the value used in the analysis. Absorbance was measured at 492 nm using a FLUOstar Omega microplate reader (BMG LABTECH GmbH, Ortenberg, Germany), with the freshly prepared XTT solution serving as the blank.

### 4.7. Scanning Electron Microscopy (SEM)

Surface topography was analyzed by scanning electron microscopy (SEM) using a JSM-7500F microscope (JEOL, Tokyo, Japan). The specimens were carbon-coated before imaging, and representative micrographs were obtained at a magnification of 1000×; one micrograph (panel D of Figure 11) was obtained at 1200×.

### 4.8. Experimental Design and Statistical Analysis

For biofilm formation, the quantitative outcome variables were colony-forming unit counts per milliliter (CFU/mL) and absorbance values obtained from the XTT assay (cell metabolic activity). These variables were analyzed according to two experimental factors: resin material (three levels) and disinfectant solution (five levels).

Data were analyzed by two-way analysis of variance (ANOVA), with resin material and disinfectant solution as factors, including the interaction term. CFU counts were analyzed as log_10_(CFU/mL) and XTT results as log-transformed optical density; XTT data are presented as a percentage of the corresponding resin control. Multiple comparisons were performed using the Tukey test. All statistical analyses were conducted using a significance level of 5% (α = 0.05). Analyses were performed in jamovi (version 2.6; The jamovi project, Sydney, Australia; https://www.jamovi.org) and GraphPad Prism (version 11; GraphPad Software, Boston, MA, USA; https://www.graphpad.com). Microbiological assays were performed on three independent occasions, with two specimens per experimental condition assigned to each assay on each occasion, resulting in *n* = 6 specimens per experimental condition for both the CFU/mL counts and the XTT assay. For the XTT assay, each specimen was read in duplicate aliquots and the two readings were averaged before analysis, so that the experimental unit is the specimen in both assays. As a sensitivity analysis, both assays were additionally analysed at the level of the independent experimental occasion (*n* = 3), which reproduced every effect reported. For SEM analysis, one additional independent experiment was performed in duplicate, resulting in *n* = 2 specimens per experimental condition.

The nanomechanical characterization obtained by atomic force microscopy, comprising surface roughness, Young’s modulus and the single-cell adhesion parameters, is reported descriptively. Distributions are described by the median and the range or interquartile range and are presented as frequency histograms. No inferential comparison is made between materials for these outcomes.

For the single-cell force spectroscopy, the experimental unit is the individual cell probe (*n* = 4), each contacted with all three surfaces in a paired design; the force curves acquired with a given probe on a given surface are technical replicates of that probe and are used to describe its distribution, not as independent observations. For surface roughness (Ra and RMS, nm) and Young’s modulus (GPa), the replicates are the four quantitative-imaging maps acquired per material, each map being summarised by the median of its indentation points.

No a priori sample size calculation was performed for the atomic force microscopy outcomes. The numbers of replicates used, four quantitative-imaging maps and four independent cell probes per material, were defined before the analysis and are consistent with those commonly employed in AFM-based nanomechanical and single-cell force spectroscopy studies, in which each cell probe itself yields hundreds of individual force curves [43,44,56,57,69]. Because this number of replicates does not support formal comparison between materials, all atomic force microscopy outcomes are reported descriptively, as stated above.

For microbiological outcomes, comparisons among the three resins and the five disinfectant solutions were performed as described above, with a significance level of 5% (alpha = 0.05). The number of observations entering each comparison was the same for both assays. Each model was fitted to 90 observations, corresponding to the fifteen experimental conditions with six specimens each, and had 75 residual degrees of freedom. Comparisons between two experimental conditions therefore contrast 6 specimens per group, comparisons between two disinfectant solutions contrast 18 per group, and comparisons between two resins contrast 30 per group. The exact *p* value of every comparison discussed in the Results is reported in the running text of that section. Analyses were performed using the software specified above.

Distribution histograms are presented in terms of relative frequency (%), with a log-normal fit line showing the central tendency.

Qualitative data obtained from SEM were analyzed descriptively based on representative micrographs for each experimental condition.

## 5. Conclusions

The 3D-printed resin exhibited the highest surface roughness, and the three resins differed in stiffness. The 3D-printed resin was more susceptible to microbial colonization than the milled resin and more resistant to the effect of the disinfectants. Since this is an in vitro and short-term study, the disinfectant effectiveness of chlorhexidine in reducing CFU counts should be interpreted under the experimental conditions tested. All disinfectant solutions promoted a reduction in biofilm metabolic activity, except PHMB for the 3D-printed occlusal splint resin, where the highest surface roughness, filamentous morphology of *C. albicans*, and high metabolic activity were found, indicating that the response to the disinfectant is substrate-dependent. The single-cell adhesion distributions, which are reported descriptively, were consistent with these findings.

## Figures and Tables

**Figure 1:**
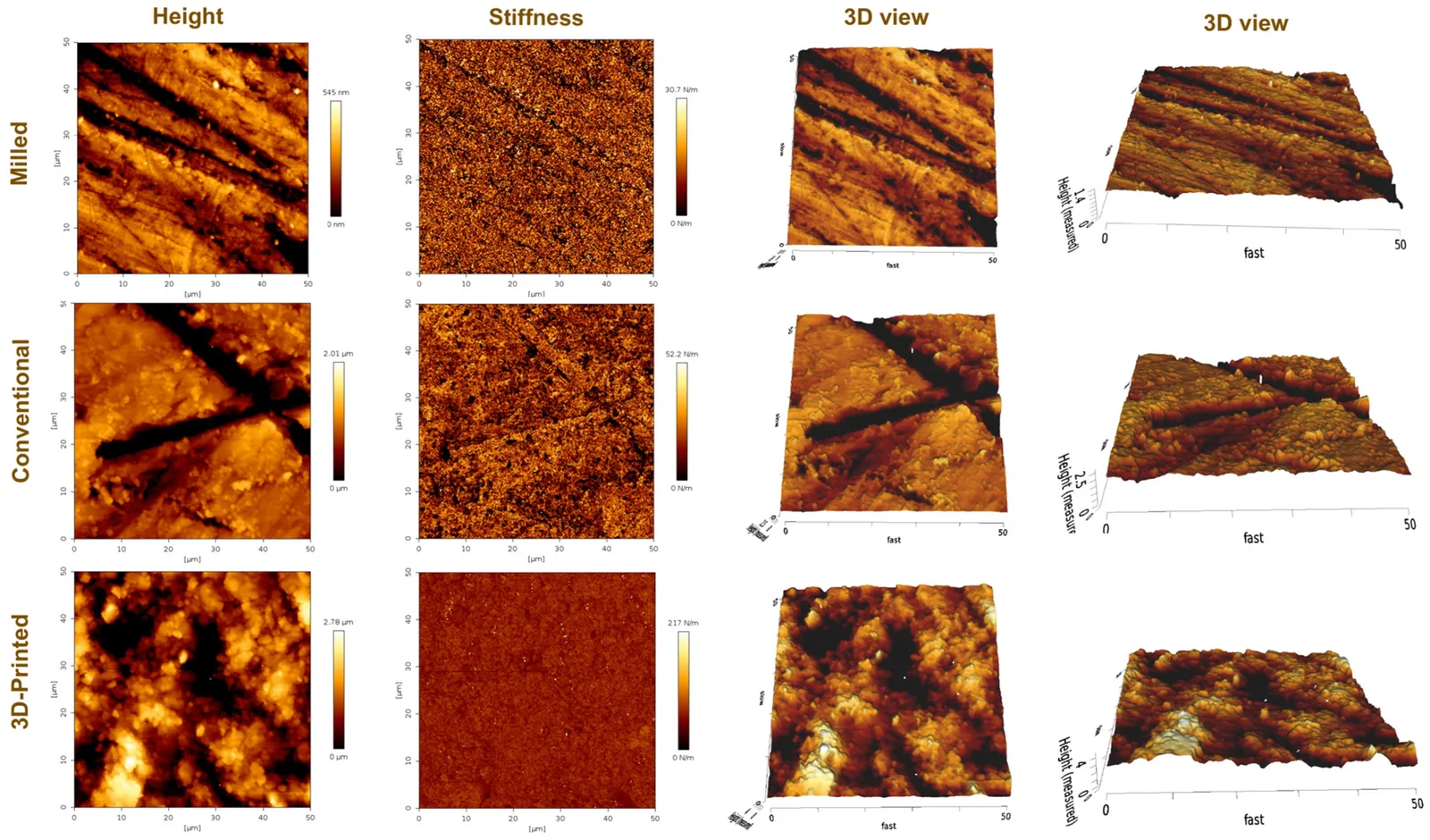
Topography and stiffness maps for the three resin splint materials. Height, stiffness, and 3D reconstructions for 256 × 256 pixel maps obtained with AFM advanced quantitative imaging mode (QI).

**Figure 2:**
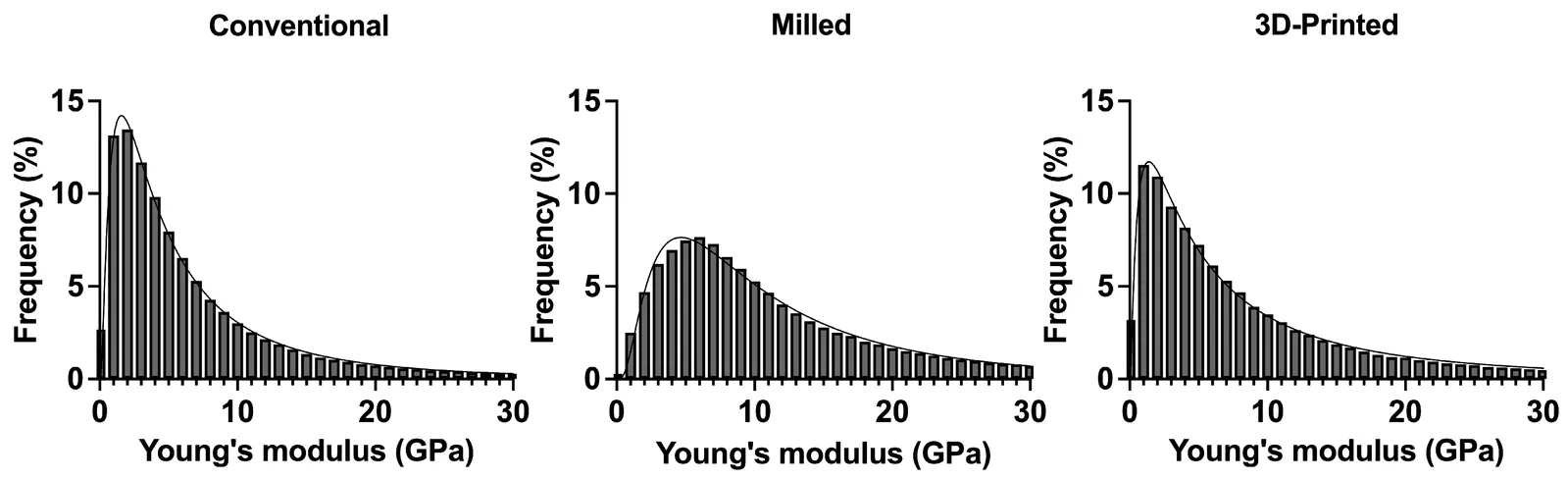
Frequency histogram of Young’s modulus (all points) by surface, with a log-normal fit curve.

**Figure 3:**
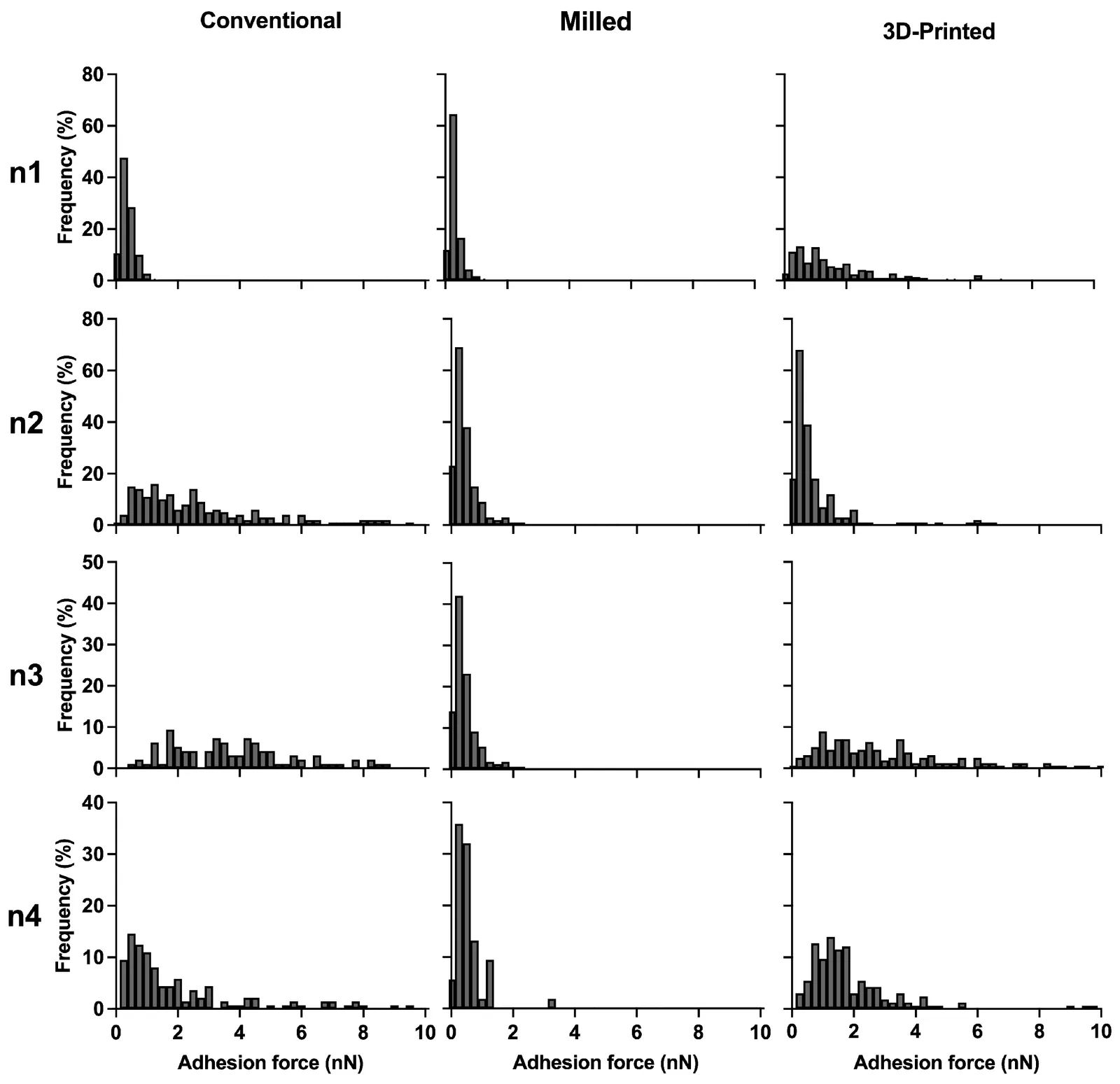
Frequency histogram of the maximum adhesion force (nN) per force curve. Rows n1 to n4 are the four cell probes, and the columns are the conventional, milled and 3D-printed resins.

**Figure 4:**
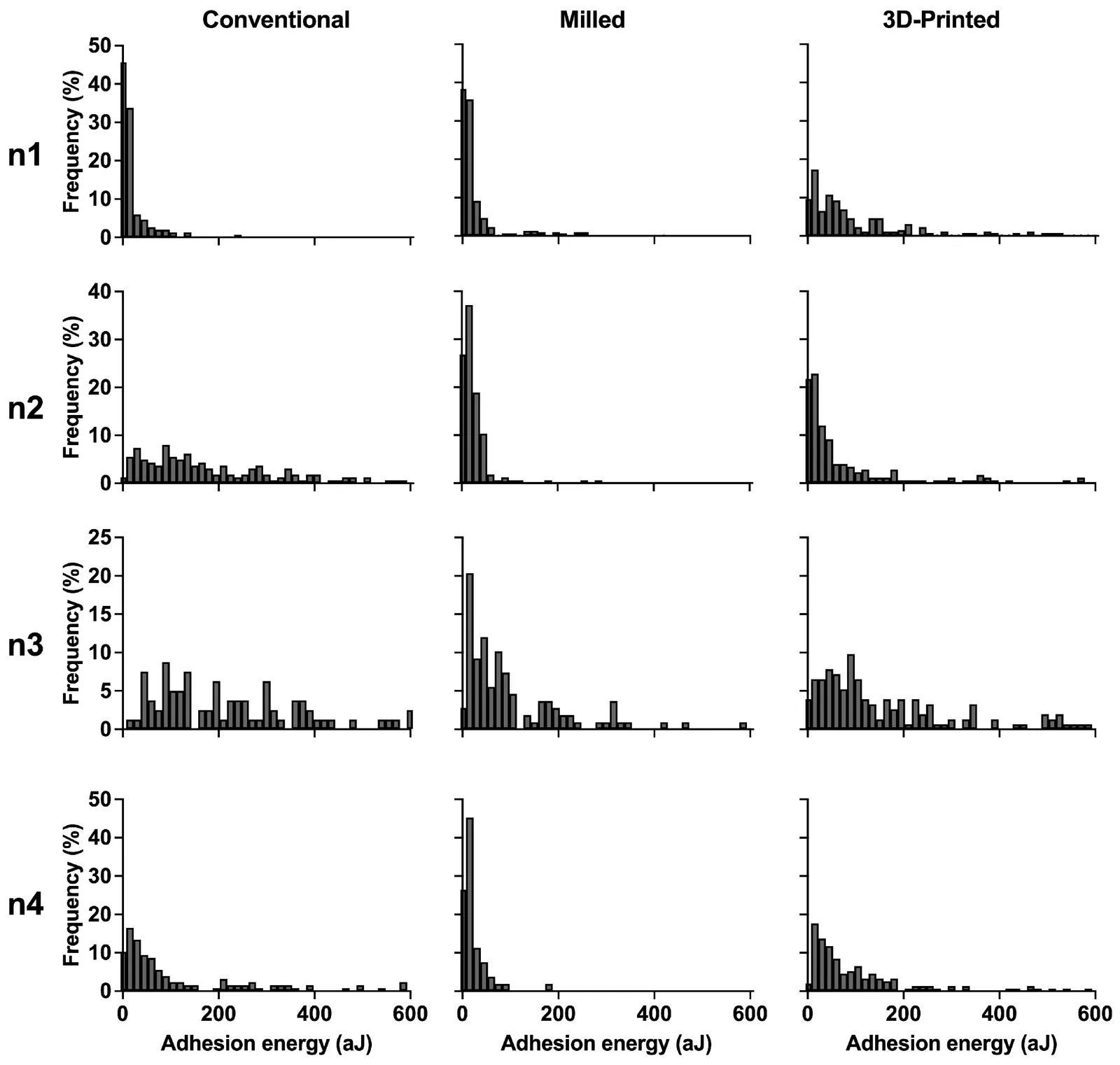
Frequency histogram of the adhesion energy (aJ) per force curve. Rows n1 to n4 are the four cell probes, and the columns are the conventional, milled and 3D-printed resins.

**Figure 5:**
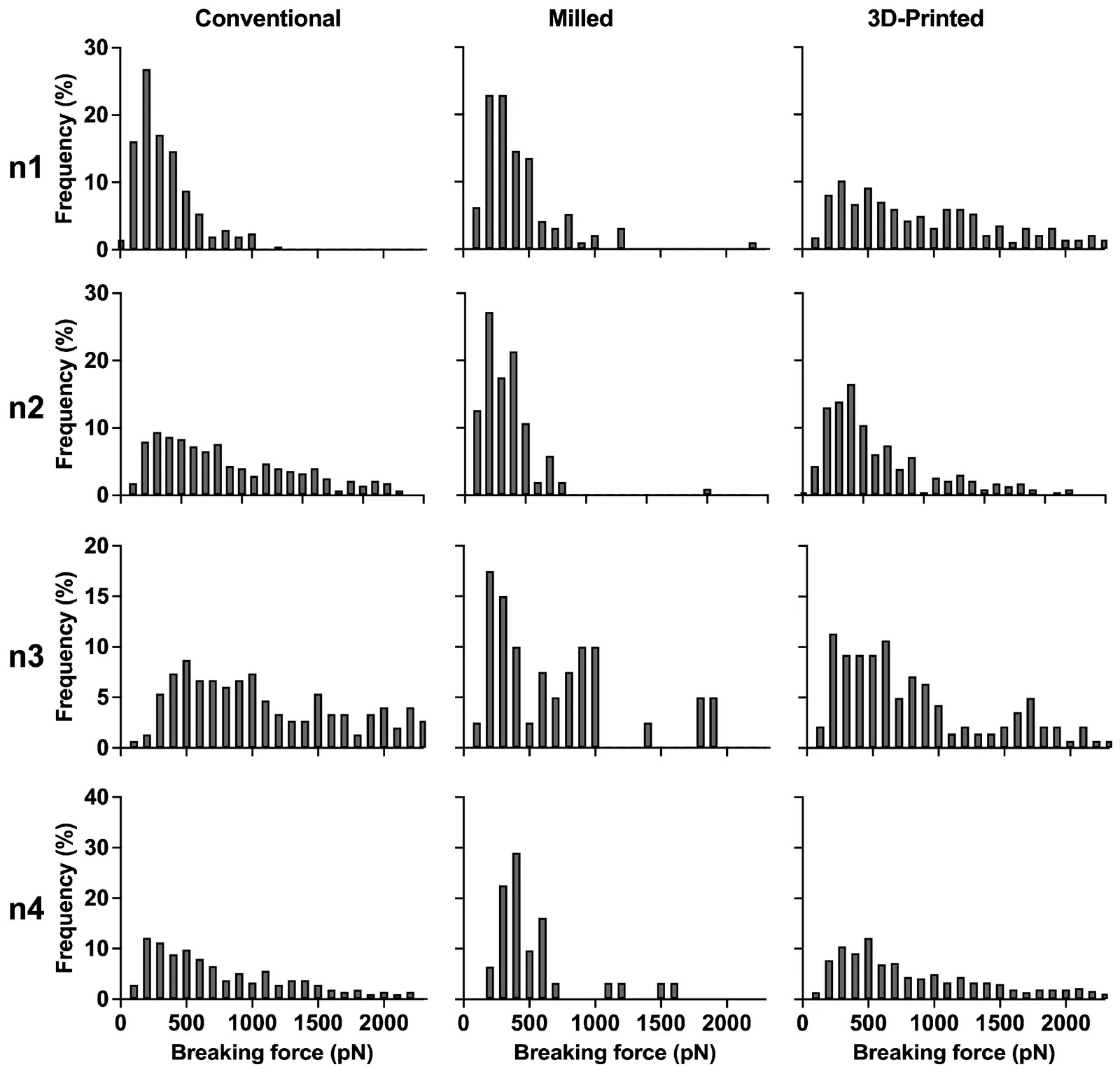
Frequency histogram of the breaking force (pN) of the individual unbinding events. Rows n1 to n4 are the four cell probes, and the columns are the conventional, milled and 3D-printed resins.

**Figure 6:**
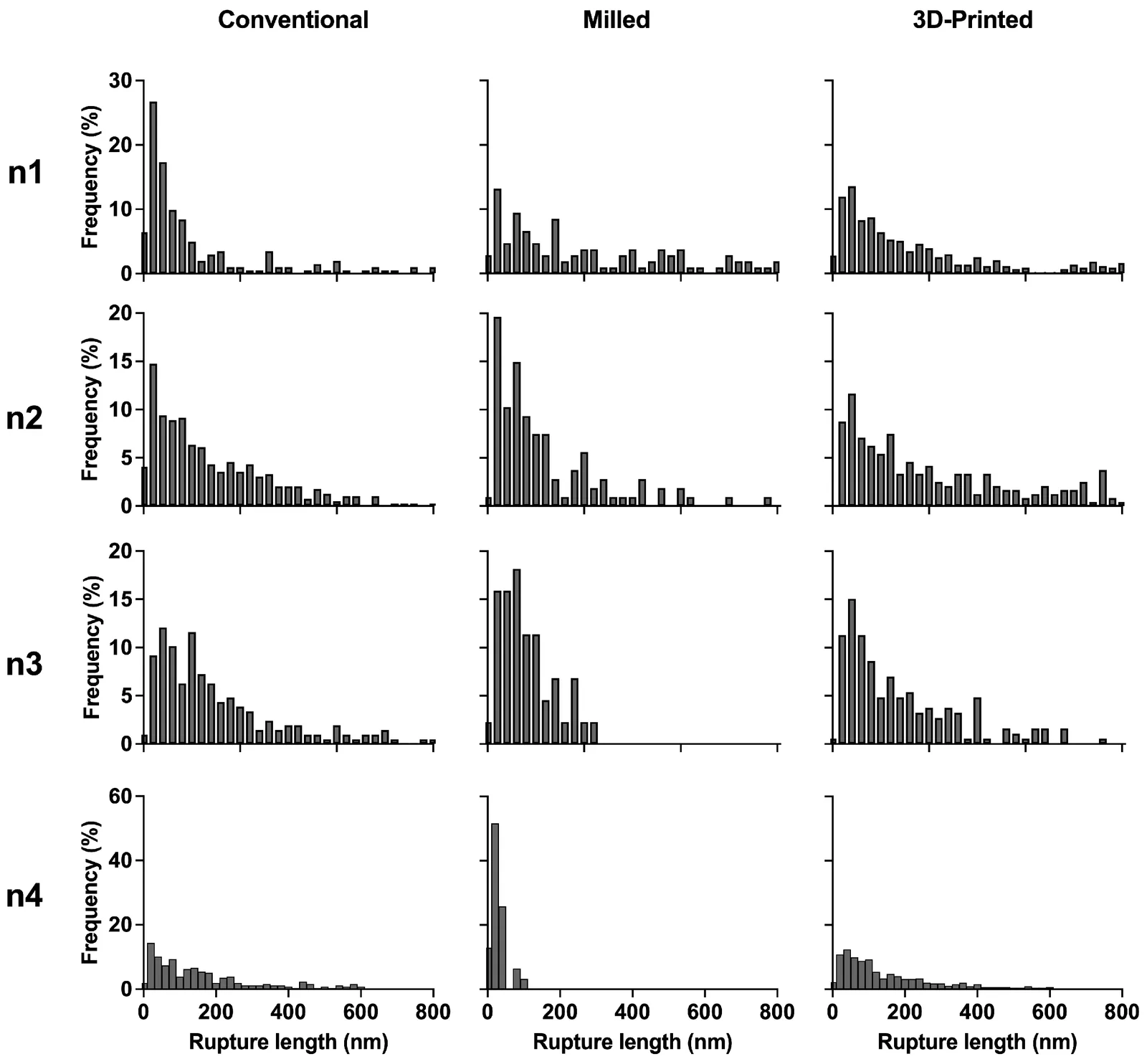
Frequency histogram of the rupture length (nm) of the individual unbinding events. Rows n1 to n4 are the four cell probes, and the columns are the conventional, milled and 3D-printed resins.

**Figure 7:**
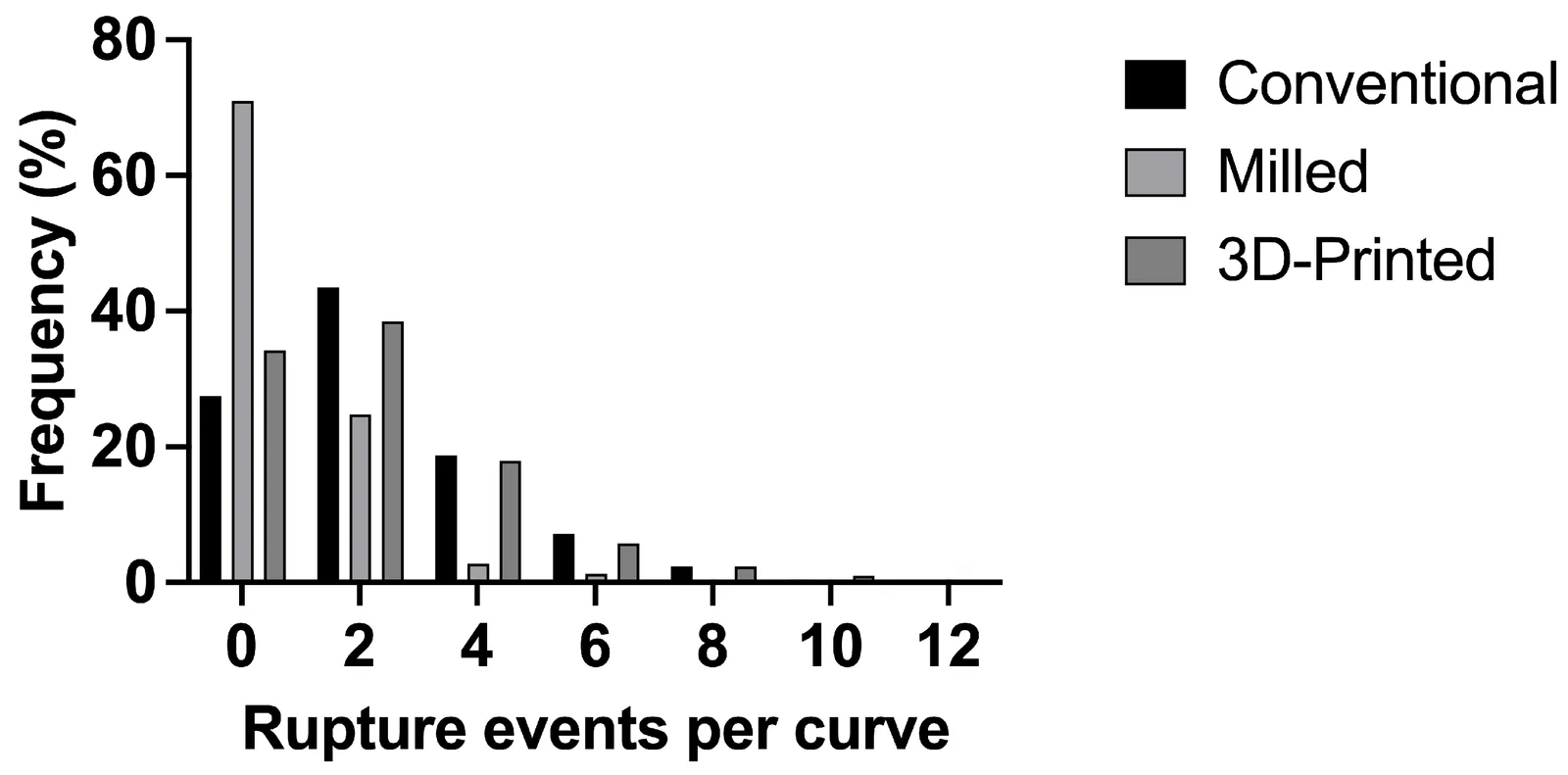
Frequency histogram of the number of rupture events per curve for each resin.

**Figure 8:**
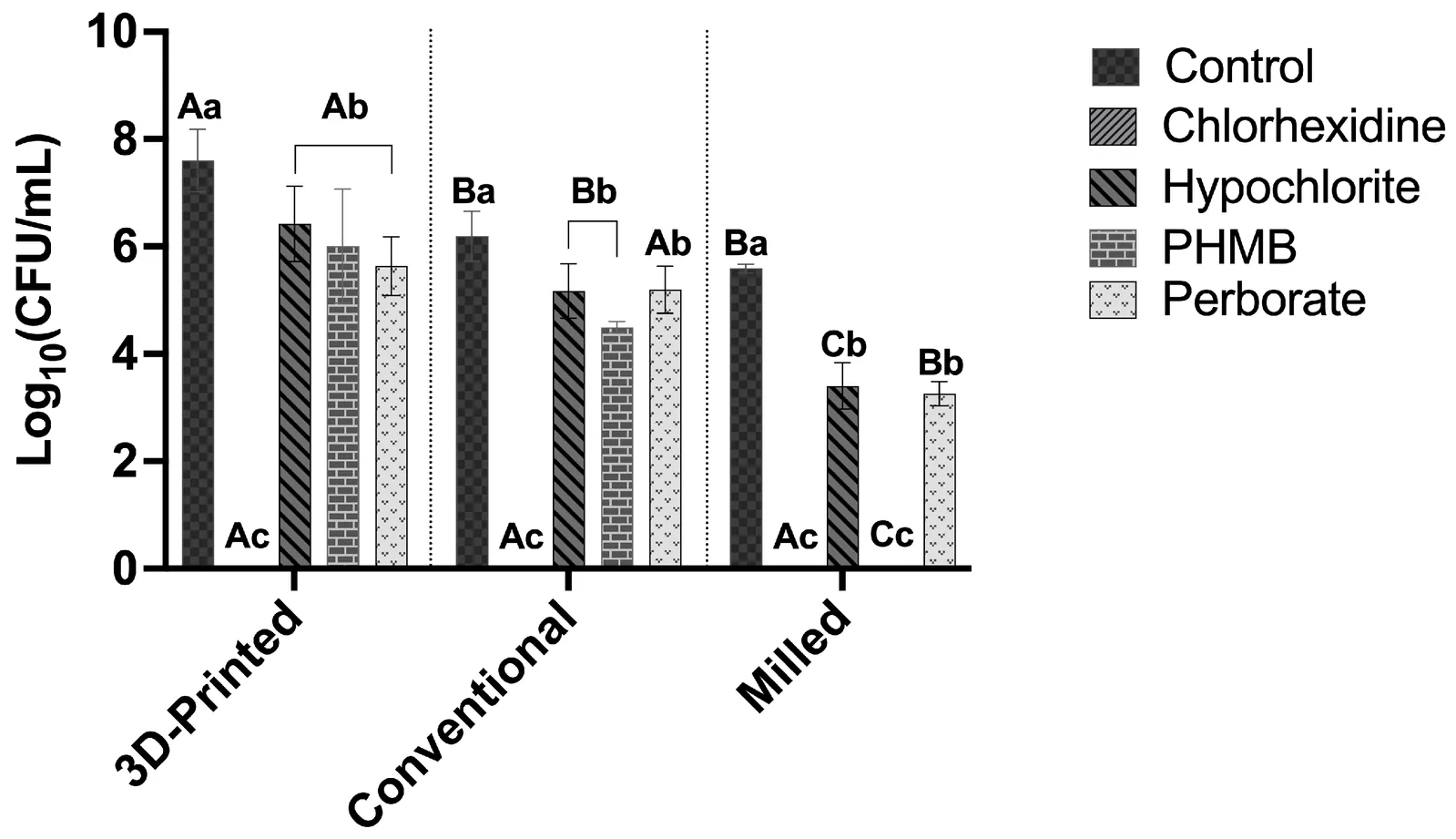
Microbial load [log_10_(CFU/mL)] by resin and disinfectant (mean and 95% CI, *n* = 6). Lowercase letters compare disinfectants within each resin. Uppercase letters compare resins within each disinfectant. Groups sharing the same letter do not differ (Tukey, *p* > 0.05). These characters are statistical grouping symbols and not panel identifiers. For example, “Aa” on the control bar of the 3D-printed resin means that this bar belongs to group A when the three resins are compared within the control condition, and to group a when the five disinfectants are compared within that resin. A bracket spanning two or more bars indicates that the letters shown above it apply to every bar under it.

**Figure 9:**
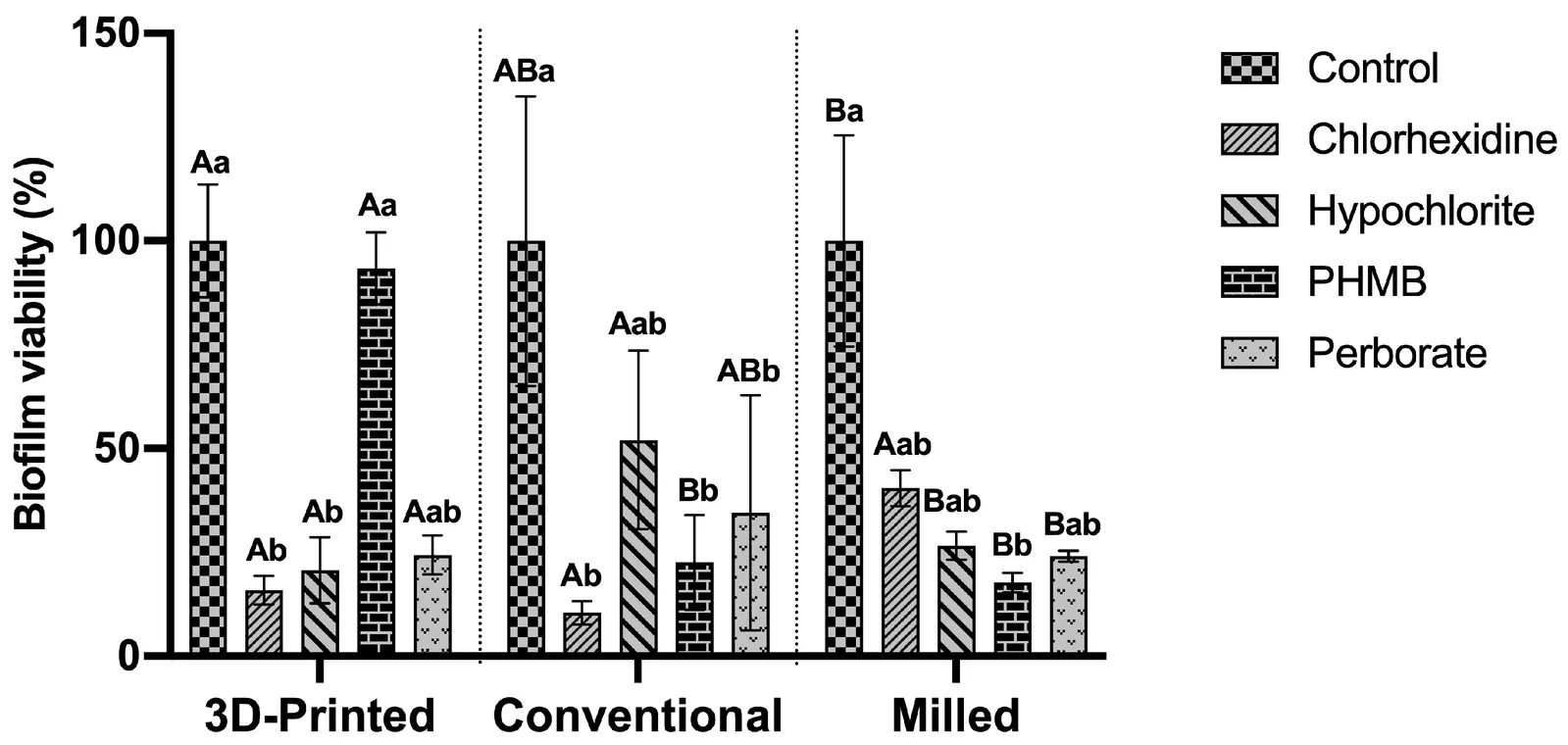
Biofilm viability (XTT), expressed as a percentage of the control of the corresponding resin (mean ± SEM, *n* = 6 specimens per experimental condition). Dotted line = control (100%). Bars sharing the same letter did not differ significantly (two-way ANOVA followed by Tukey’s test comparing all cell means, *p* > 0.05). Uppercase letters compare resins within a given disinfectant and lowercase letters compare disinfectants within a given resin; these characters are statistical grouping symbols and not panel identifiers.

**Figure 10:**
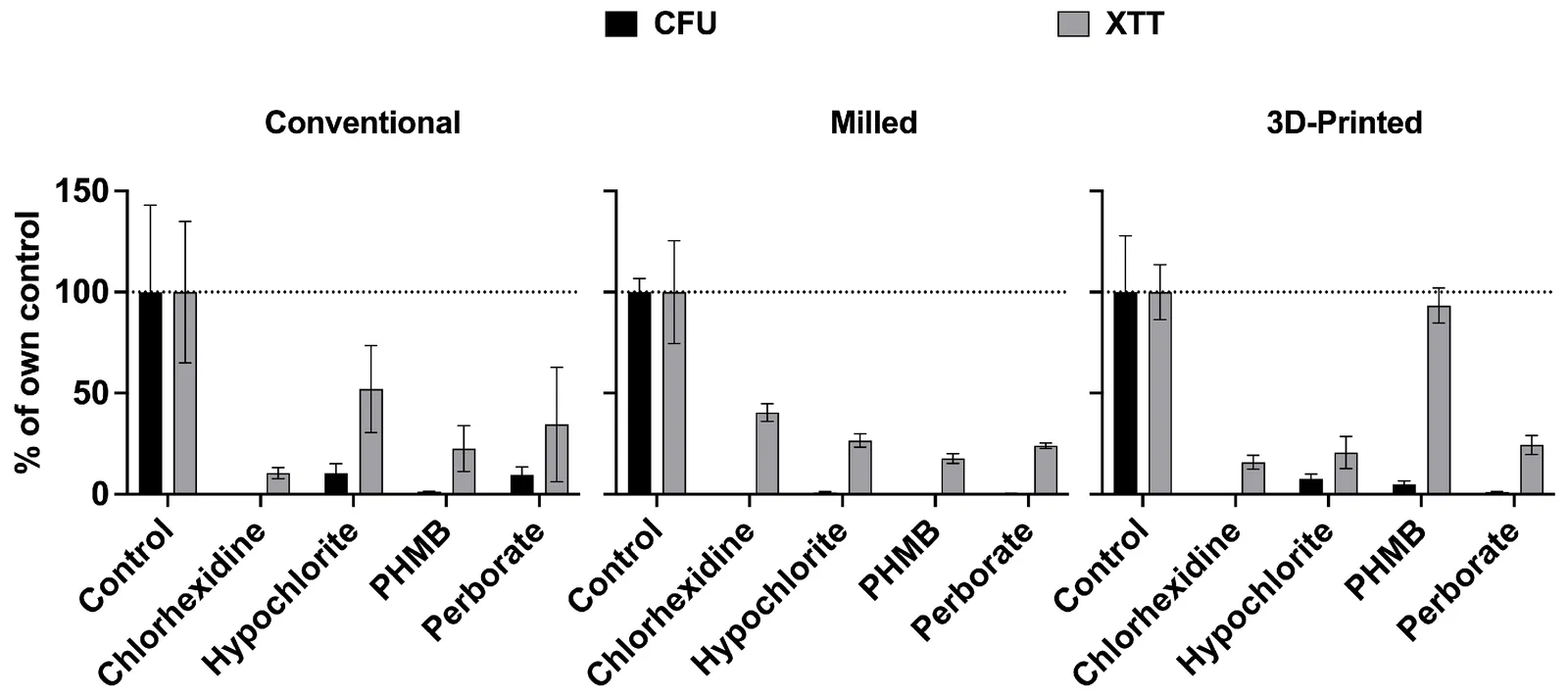
Comparison between cultivable load (CFU) and biofilm metabolic activity (XTT), by resin, as a percentage (%) of the control of the corresponding resin. Bars = mean ± SE (CFU *n* = 6; XTT *n* = 6). Black bars = CFU; grey bars = XTT. Dotted line = control (100%).

**Figure 11:**
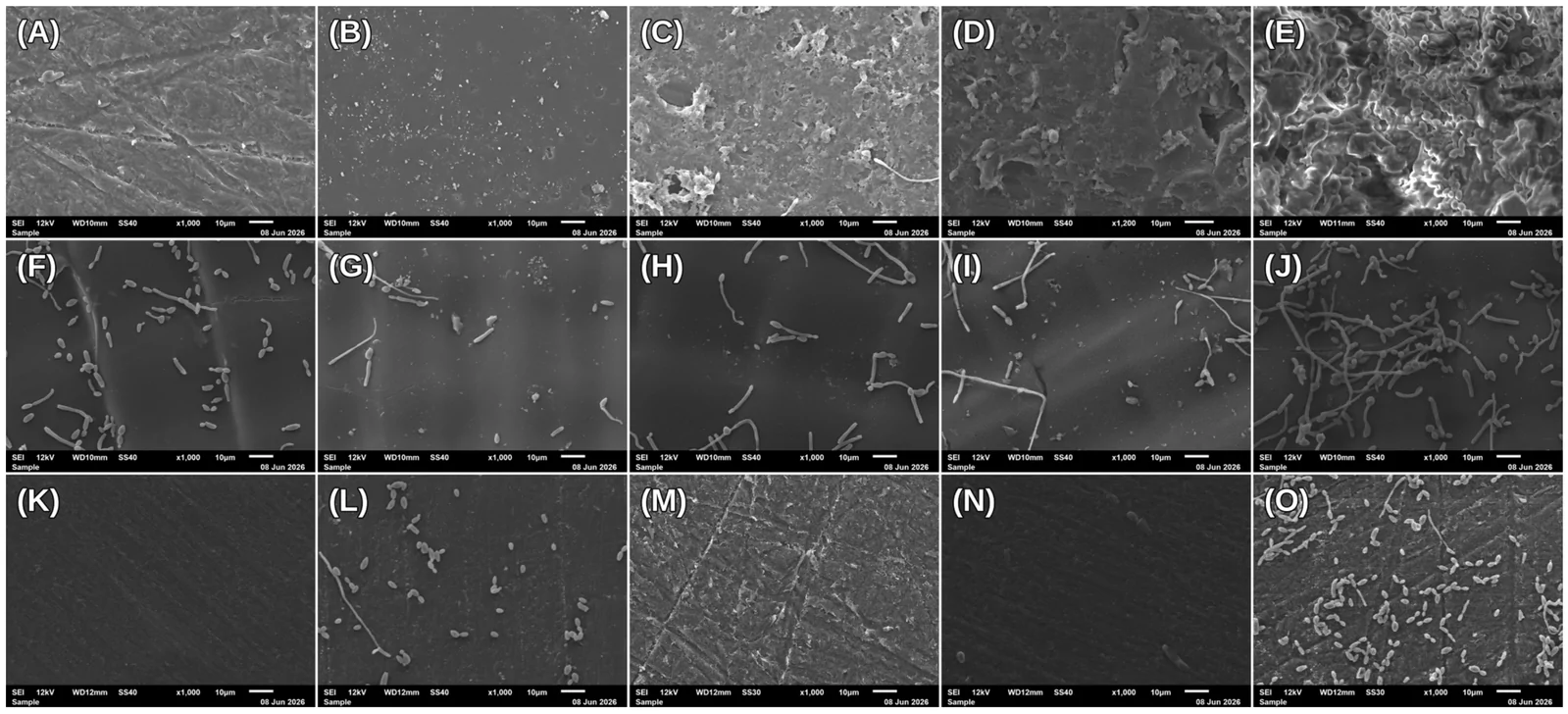
Scanning electron microscopy (SEM) images of the Candida albicans biofilm on conventional, 3D-printed, and milled resins after treatment with different disinfectant solutions. (**A**–**E**) conventional; (**F**–**J**) 3D-printed; (**K**–**O**) milled. Chlorhexidine (**A**,**F**,**K**); Corega Tabs (**B**,**G**,**L**); sodium hypochlorite (**C**,**H**,**M**); PHMB (**D**,**I**,**N**); and PBS, control (**E**,**J**,**O**). Micrographs were acquired at 1000×, except panel D (PHMB, conventional resin), which was acquired at 1200×.

## Data Availability

The original contributions presented in this study are included in the article/Supplementary Materials. Further inquiries can be directed to the corresponding author.

## Supplementary Materials

The following supporting information can be downloaded at https://www.mdpi.com/article/10.3390/antibiotics15100941/s1. Figure S1: Frequency histogram of maximum adhesion forces (nN) between *C. albicans* functionalized AFM probes and acrylic surfaces. Figure S2: Frequency histogram of adhesion energy (aJ), with a log-normal fit curve. Figure S3: Frequency histogram of breaking force (pN) per surface, with a log-normal fit curve. Figure S4: Frequency histogram of rupture length (nm) by surface, with a log-normal fit curve.

## Abbreviations

The following abbreviations are used in this manuscript:

PHMB	Polyhexamethylene biguanide
PBS	Phosphate-buffered saline
CFU	Colony-forming unit
SEM	Scanning electron microscopy
AFM	Atomic force microscopy
SCFS	Single-cell force spectroscopy
TMD	Temporomandibular disorders
CAD/CAM	Computer-Aided Design/Computer-Aided Manufacturing
SFE	Surface free energy
Ra	Roughness average
RMS	Root Mean Square
Rq	Roughness quadratic

## References

[1] Nassif M., Haddad C., Habli L., Zoghby A. (2023). Materials and manufacturing techniques for occlusal splints: A literature review. J. Oral Rehabil..

[2] Benli M., Eker Gümüş B., Kahraman Y., Gökçen-Rohlig B., Evlioğlu G., Huck O., Özcan M. (2020). Surface roughness and wear behavior of occlusal splint materials made of contemporary and high-performance polymers. Odontology.

[3] Fricton J., Look J.O., Wright E., Alencar F.G.P.J., Chen H., Lang M., Ouyang W., Velly A.M. (2010). Systematic review and meta-analysis of randomized controlled trials evaluating intraoral orthopedic appliances for temporomandibular disorders. J. Orofac. Pain.

[4] Gil-Martínez A., Paris-Alemany A., López-de-Uralde-Villanueva I., La Touche R. (2018). Management of pain in patients with temporomandibular disorder (TMD): Challenges and solutions. J. Pain Res..

[5] Proff P., Richter E.-J., Blens T., Fanghänel J., Hützen D., Kordass B., Gedrange T., Rottner K. (2007). A michigan-type occlusal splint with spring-loaded mandibular protrusion functionality for treatment of anterior disk dislocation with reduction. Ann. Anat..

[6] Šimunović L., Čimić S., Meštrović S. (2025). Three-Dimensionally Printed Splints in Dentistry: A Comprehensive Review. Dent. J..

[7] Aslanimehr M., Mojarad N., Ranjbar S., Aalaei S. (2018). In vitro comparison of the effects of microwave irradiation and chemical and mechanical methods on the disinfection of complete dentures contaminated with Candida albicans. Dent. Res. J..

[8] Wang S.M., Li Z., Wang G.B., Ye H.Q., Liu Y.S., Tong D., Gao W.H., Zhou Y.S. (2019). Preliminary clinical application of complete digital workflow of design and manufacturing occlusal splint for sleep bruxism. Beijing Da Xue Xue Bao Yi Xue Ban.

[9] Bürgers R., Schneider-Brachert W., Rosentritt M., Handel G., Hahnel S. (2009). Candida albicans adhesion to composite resin materials. Clin. Oral Investig..

[10] Kolenbrander P.E., London J. (1993). Adhere today, here tomorrow: Oral bacterial adherence. J. Bacteriol..

[11] Ruby J., Barbeau J. (2002). The Buccale Puzzle: The Symbiotic Nature of Endogenous Infections of the Oral Cavity. Can. J. Infect. Dis. Med. Microbiol..

[12] Wuersching S.N., Westphal D., Stawarczyk B., Edelhoff D., Kollmuss M. (2023). Surface properties and initial bacterial biofilm growth on 3D-printed oral appliances: A comparative in vitro study. Clin. Oral Investig..

[13] Baraniya D., Chen T., Nahar A., Alakwaa F., Hill J., Tellez M., Ismail A., Puri S., Al-Hebshi N.N. (2020). Supragingival mycobiome and inter-kingdom interactions in dental caries. J. Oral Microbiol..

[14] Finkel J.S., Mitchell A.P. (2011). Genetic control of Candida albicans biofilm development. Nat. Rev. Microbiol..

[15] Douglas L.J. (2003). Candida biofilms and their role in infection. Trends Microbiol..

[16] Akpan A., Morgan R. (2002). Oral candidiasis. Postgrad. Med. J..

[17] Sánchez-Vargas L.O., Estrada-Barraza D., Pozos-Guillen A.J., Rivas-Caceres R. (2013). Biofilm formation by oral clinical isolates of Candida species. Arch. Oral Biol..

[18] Chadwick P.R. (1999). Revised UK guidelines for the control of methicillin-resistant Staphylococcus aureus in hospitals. Int. J. Antimicrob. Agents.

[19] Koo H., Andes D.R., Krysan D.J. (2018). Candida–streptococcal interactions in biofilm-associated oral diseases. PLoS Pathog..

[20] Kim H.-E., Liu Y., Dhall A., Bawazir M., Koo H., Hwang G. (2021). Synergism of Streptococcus mutans and Candida albicans Reinforces Biofilm Maturation and Acidogenicity in Saliva: An In Vitro Study. Front. Cell. Infect. Microbiol..

[21] Falsetta M.L., Klein M.I., Colonne P.M., Scott-Anne K., Gregoire S., Pai C.-H., Gonzalez-Begne M., Watson G., Krysan D.J., Bowen W.H. (2014). Symbiotic Relationship between Streptococcus mutans and Candida albicans Synergizes Virulence of Plaque Biofilms In Vivo. Infect. Immun..

[22] Minagi S., Miyake Y., Inagaki K., Tsuru H., Suginaka H. (1985). Hydrophobic interaction in Candida albicans and Candida tropicalis adherence to various denture base resin materials. Infect. Immun..

[23] Lee M.-J., Kim M.-J., Oh S.-H., Kwon J.-S. (2020). Novel Dental Poly (Methyl Methacrylate) Containing Phytoncide for Antifungal Effect and Inhibition of Oral Multispecies Biofilm. Materials.

[24] Revilla-León M., Meyers M.J., Zandinejad A., Özcan M. (2019). A review on chemical composition, mechanical properties, and manufacturing work flow of additively manufactured current polymers for interim dental restorations. J. Esthet. Restor. Dent..

[25] Papadiochou S., Polyzois G. (2018). Hygiene practices in removable prosthodontics: A systematic review. Int. J. Dent. Hyg..

[26] de Carvalho B.M.D.F., Garcia B.A., Gomes A.K.P., Alcantara D.D., de Freitas Pontes K.M. (2023). Antimicrobial Photodynamic Therapy as a Technique for Decontamination of Acrylic Resin Devices Provided by Different Dental Laboratories. J. Lasers Med. Sci..

[27] Buergers R., Rosentritt M., Schneider-Brachert W., Behr M., Handel G., Hahnel S. (2008). Efficacy of denture disinfection methods in controlling Candida albicans colonization in vitro. Acta Odontol. Scand..

[28] Pavarina A.C., Machado A.L., Giampaolo E.T., Vergani C.E. (2003). Effects of chemical disinfectants on the transverse strength of denture base acrylic resins. J. Oral Rehabil..

[29] Pellizzaro D., Polyzois G., Machado A.L., Giampaolo E.T., Sanitá P.V., Vergani C.E. (2012). Effectiveness of mechanical brushing with different denture cleansing agents in reducing in vitro Candida albicans biofilm viability. Braz. Dent. J..

[30] Arruda C.N.F., Sorgini D.B., Oliveira V.d.C., Macedo A.P., Lovato C.H.S., Paranhos H.d.F.O. (2015). Effects of Denture Cleansers on Heat-Polymerized Acrylic Resin: A Five-Year-Simulated Period of Use. Braz. Dent. J..

[31] Badaró M.M., Bueno F.L., Arnez R.M., Oliveira V.d.C., Macedo A.P., de Souza R.F., Paranhos H.d.F.O., Silva-Lovato C.H. (2020). The effects of three disinfection protocols on Candida spp., denture stomatitis, and biofilm: A parallel group randomized controlled trial. J. Prosthet. Dent..

[32] Nunes T.S.B.S., Silva M.D.D.D., Coelho S.R.G., Viotto H.E.D.C., Pero A.C. (2023). Effectiveness of disinfectant solutions associated or not with brushing on the biofilm control of a 3D printed-denture base resin. J. Appl. Oral Sci..

[33] Mayta B.S., Candido L.A., Zancopé K., Simamoto Júnior P.C., Mendonça G., Das Neves F.D. (2025). Effect of preservation on the mechanical properties of 3D printing resins for occlusal splints. Braz. Oral Res..

[34] Chen Y.D., Shu C., Duan Z.H., Xu J.J., Li X.J., Chen F., Luo Q.J., Li X.D. (2021). Synthesis and characterization of an anti-caries and remineralizing fluorine-containing cationic polymer PHMB-F. Biomater. Sci..

[35] Mikić I.M., Cigić L., Kero D., Govorko D.K., Mehičić G.P., Andrašević A.T., Simeon P. (2018). Antimicrobial effectiveness of polyhexamethylene biguanide on Enterococcus faecalis, Staphylococcus epidermidis and Candida albicans. Med. Glas..

[36] Jayanandan M., Veeraraghavan V.P., Govindarajan S., Subramani S.M. (2026). Development of oral dysbiosis following use of antimicrobial mouthwashes: A systematic review. Odontology.

[37] Khan A., Nikhil V., Pandey A., Chaturvedi P. (2022). Effectiveness of polyhexamethylene biguanide, chlorhexidine, and calcium hydroxide intracanal medicament against intraradicular mature polymicrobial biofilm: A microbiological study. J. Conserv. Dent..

[38] Khabadze Z., Generalova Y., Kulikova A., Podoprigora I., Abdulkerimova S., Bakaev Y., Makeeva M., Dashtieva M., Balashova M., Gadzhiev F. (2023). Irrigation in Endodontics: Polyhexanide Is a Promising Antibacterial Polymer in Root Canal Treatment. Dent. J..

[39] Aguayo S., Marshall H., Pratten J., Bradshaw D., Brown J.S., Porter S.R., Spratt D., Bozec L. (2017). Early Adhesion of *Candida albicans* onto Dental Acrylic Surfaces. J. Dent. Res..

[40] Turanoglu O.F., Cevlik E.T., Vural C. (2024). Investigation of adhesion status of Candida species to the surface of resin materials produced at different angles with additive manufacturing. BMC Oral Health.

[41] Brose L., Tamjidtash S., Doll-Nikutta K., Stiesch M., Kommerein N., Hahnel S. (2026). Influence of roughness and surface free energy on adhesion and biofilm formation of Candida albicans to polymeric materials. Res. Sq..

[42] Mouhat M., Moorehead R., Murdoch C. (2020). In vitro *Candida albicans* biofilm formation on different titanium surface topographies. Biomater. Investig. Dent..

[43] Schuh C.M.A.P., Benso B., Naulin P.A., Barrera N.P., Bozec L., Aguayo S. (2021). Modulatory Effect of Glycated Collagen on Oral Streptococcal Nanoadhesion. J. Dent. Res..

[44] Cho D.H., Aguayo S., Cartagena-Rivera A.X. (2023). Atomic force microscopy-mediated mechanobiological profiling of complex human tissues. Biomaterials.

[45] Deptuła P., Chmielewska-Deptuła S., Spałek J., Kaliniak S., Okła S., Bucki R. (2026). Decoding the pathogenesis of Candida albicans infection and evaluating the efficacy of antifungal drugs using atomic force microscopy. Biomed. Pharmacother..

[46] Valentini F., Luz M.S., Boscato N., Pereira-Cenci T. (2013). Biofilm formation on denture liners in a randomised controlled in situ trial. J. Dent..

[47] de Freitas R.F.C.P., Duarte S., Feitosa S., Dutra V., Lin W.-S., Panariello B.H.D., Carreiro A.d.F.P. (2023). Physical, Mechanical, and Anti-Biofilm Formation Properties of CAD-CAM Milled or 3D Printed Denture Base Resins: In Vitro Analysis. J. Prosthodont..

[48] Alfouzan A.F., Tuwaym M., Aldaghri E.N., Alojaymi T., Alotiabi H.M., Taweel S.M.A., Al-Otaibi H.N., Ali R., Alshehri H., Labban N. (2023). Efficacy of Denture Cleansers on Microbial Adherence and Surface Topography of Conventional and CAD/CAM-Processed Denture Base Resins. Polymers.

[49] Schubert A., Bürgers R., Baum F., Kurbad O., Wassmann T. (2021). Influence of the Manufacturing Method on the Adhesion of Candida albicans and Streptococcus mutans to Oral Splint Resins. Polymers.

[50] Sahin M.A., Yenidunya O.G., Kaleli I., Atca M. (2025). Surface roughness and biofilm formation on tooth-colored restorative materials immersed in food-simulating liquids. BMC Oral Health.

[51] Zheng S., Bawazir M., Dhall A., Kim H.-E., He L., Heo J., Hwang G. (2021). Implication of Surface Properties, Bacterial Motility, and Hydrodynamic Conditions on Bacterial Surface Sensing and Their Initial Adhesion. Front. Bioeng. Biotechnol..

[52] Ferro A.C., De Oliveira J.S., Scabelo L., Araújo L.V., Mota C., Baker M.B., Jorge J.H. (2026). Effect of surface polishing on roughness, biofilm formation, and biocompatibility of LCD-printed denture base polymer. Sci. Rep..

[53] Zissis A.J., Polyzois G.L., Yannikakis S.A., Harrison A. (2000). Roughness of denture materials: A comparative study. Int. J. Prosthodont..

[54] Li P., Fernandez P.K., Spintzyk S., Schmidt F., Beuer F., Unkovskiy A. (2022). Effect of additive manufacturing method and build angle on surface characteristics and Candida albicans adhesion to 3D printed denture base polymers. J. Dent..

[55] Shim J.S., Kim J.-E., Jeong S.H., Choi Y.J., Ryu J.J. (2020). Printing accuracy, mechanical properties, surface characteristics, and microbial adhesion of 3D-printed resins with various printing orientations. J. Prosthet. Dent..

[56] Jones C.R., Zhang Z.J., Tsai H.-J., Drummond R.A. (2023). Quantifying the Mechanical Properties of Yeast Candida albicans Using Atomic Force Microscopy-based Force Spectroscopy. Antifungal Immunity.

[57] Vitry P., Valotteau C., Feuillie C., Bernard S., Alsteens D., Geoghegan J.A., Dufrêne Y.F. (2017). Force-Induced Strengthening of the Interaction between Staphylococcus aureus Clumping Factor B and Loricrin. mBio.

[58] Mohammadi Z., Abbott P.V. (2009). The properties and applications of chlorhexidine in endodontics. Int. Endod. J..

[59] Zaugg L.K., Zitzmann N.U., Hauser-Gerspach I., Waltimo T., Weiger R., Krastl G. (2014). Antimicrobial activity of short- and medium-term applications of polyhexamethylene biguanide, chlorhexidine digluconate and calcium hydroxide in infected immature bovine teeth in vitro. Dent. Traumatol..

[60] Gulati M., Nobile C.J. (2016). Candida albicans biofilms: Development, regulation, and molecular mechanisms. Microbes Infect..

[61] Sowlati-Hashjin S., Carbone P., Karttunen M. (2020). Insights into the Polyhexamethylene Biguanide (PHMB) Mechanism of Action on Bacterial Membrane and DNA: A Molecular Dynamics Study. J. Phys. Chem. B.

[62] Carvalho C.G., Câmara J.V.F., Méndez D.A.C., Schestakow A., Cruvinel T., Santos P.S.d.S. (2026). In vitro antibiofilm and anti-caries effects of polyhexamethylene biguanide under a microcosm biofilm model. J. Dent..

[63] Bento V.A.A., Sayeg J.M.C., Rosa C.D.D.R.D., Lopes L.F.d.T.P., Marques M.C.S., Pellizzer E.P. (2024). Efficacy of denture cleansers on Candida albicans adhesion and their effects on the properties of conventional, milled CAD/CAM, and 3D-printed denture bases. Clin. Oral Investig..

[64] De Souza G.B., Viotto H.E.D.C., Queiroz J.F.C., Moisés L.D.S., Coelho S.R.G., Marin D.O.M., Pero A.C. (2026). Evaluation of methods for removing stains from a 3D printed resin for denture teeth. J. Prosthet. Dent..

[65] Jagger D.C., Harrison A. (1995). Denture cleansing--the best approach. Br. Dent. J..

[66] Mühlemann S., Bernini J.M., Sener B., Hämmerle C.H., Özcan M. (2019). Effect of Aging on Stained Monolithic Resin-Ceramic CAD/CAM Materials: Quantitative and Qualitative Analysis of Surface Roughness. J. Prosthodont..

[67] da Cruz M.E.M., Oliveira J.J.R., Dovigo L.N., Fonseca R.G. (2022). Long-term effect of gastric juice alternating with brushing on the surface roughness, topography, and staining susceptibility of CAD-CAM monolithic materials. J. Prosthet. Dent..

[68] (2013). Dentistry—Base Polymers—Part 1: Denture Base Polymers.

[69] Leiva-Sabadini C., Lévesque C.M., Bozec L., Schuh C., Aguayo S. (2026). *Streptococcus mutans* Extracellular Vesicles Regulate Early *Streptococcus sanguinis* Nanoadhesion. J. Dent. Res..

[70] Gomes A.C.G., Maciel J.G., Garcia A.A.M.N., Coelho L.A.S., Rodrigues G.M., Porto V.C., Polyzois G., Klein M.I., Soares S., Urban V.M. (2024). Anti-biofilm effectiveness of protocols for cleaning complete dentures in hospitalized patients: A randomized controlled trial. J. Appl. Oral Sci..

